# Recent Advances in Wide-Bandgap Organic–Inorganic Halide Perovskite Solar Cells and Tandem Application

**DOI:** 10.1007/s40820-023-01040-6

**Published:** 2023-03-21

**Authors:** Ting Nie, Zhimin Fang, Xiaodong Ren, Yuwei Duan, Shengzhong (Frank) Liu

**Affiliations:** 1 Key Laboratory of Applied Surface and Colloid Chemistry, Ministry of Education, Shaanxi Key Laboratory for Advanced Energy Devices, Shaanxi Engineering Lab for Advanced Energy Technology, School of Materials Science and Engineering, Shaanxi Normal University, Xi’an, 710119 China; 2 Dalian National Laboratory for Clean Energy, iChEM, Dalian Institute of Chemical Physics, Chinese Academy of Sciences, Dalian, 116023 China

**Keywords:** Efficiency, Perovskite, Solar cell, Stability, Wide-bandgap

## Abstract

Perovskite-based tandem solar cells have attracted increasing interest because of its great potential to surpass the Shockley–Queisser limit set for single-junction solar cells. In the tandem architectures, the wide-bandgap (WBG) perovskites act as the front absorber to offer higher open-circuit voltage (*V*_OC_) for reduced thermalization losses. Taking advantage of tunable bandgap of the perovskite materials, the WBG perovskites can be easily obtained by substituting halide iodine with bromine, and substituting organic ions FA and MA with Cs. To date, the most concerned issues for the WBG perovskite solar cells (PSCs) are huge *V*_OC_ deficit and severe photo-induced phase separation. Reducing *V*_OC_ loss and improving photostability of the WBG PSCs are crucial for further efficiency breakthrough. Recently, scientists have made great efforts to overcome these key issues with tremendous progresses. In this review, we first summarize the recent progress of WBG perovskites from the aspects of compositions, additives, charge transport layers, interfaces and preparation methods. The key factors affecting efficiency and stability are then carefully discussed, which would provide decent guidance to develop highly efficient and stable WBG PSCs for tandem application.

## Highlights

- Wide-bandgap perovskite solar cells are reviewed in detail from the views of compositions, additives, charge transport layers, interfaces and preparation methods.
- The key factors affecting open-circuit voltage and photostability are carefully discussed.
- The future directions and challenges in developing wide-bandgap perovskite solar cells are highlighted.

## Graphical abstract

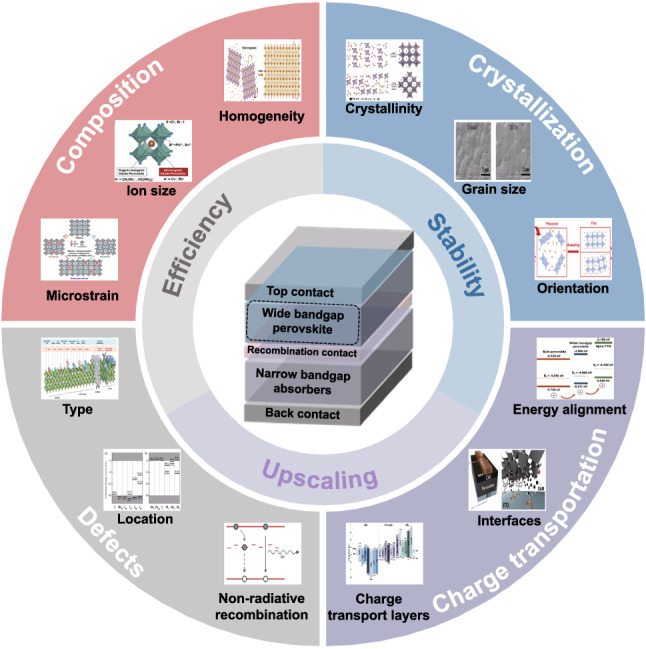

## Introduction

The development of human society is inseparable from the use of energy. However, traditional fossil energy is exhaustible and not environment friendly. Converting solar energy to electricity is one of the most promising direction to realize sustainable development. So far, the silicon (Si) solar cells are still dominating the photovoltaic (PV) market due to high efficiency, mature fabrication technology and excellent stability. Whereas, the power conversion efficiency (PCE) of silicon solar cells has reached saturation at 26.7% for years. In 2009, organic–inorganic perovskite solar cells (PSCs) came into the view of scientists around the world, since then they have become the most shining solar cells in the past decade, and the certified PCE has rapidly reached 25.7%, showing great potential for commercialization [1–7]. It is known that increasing the PCE is the most effective way to further reduce the PV module cost [8]. Impressively high PCE obtained from III–V compound semiconductors has proven the feasibility of developing multi-junction solar cells to breakthrough Shockley–Queisser (*S-Q*) limit [9–12]. Hence, it is promising to combine wide-bandgap (WBG) perovskite materials with other narrow-bandgap (NBG) light absorbers to construct tandem solar cells (TSCs) to pursue higher PCE [13–17].

**Fig. 1:**
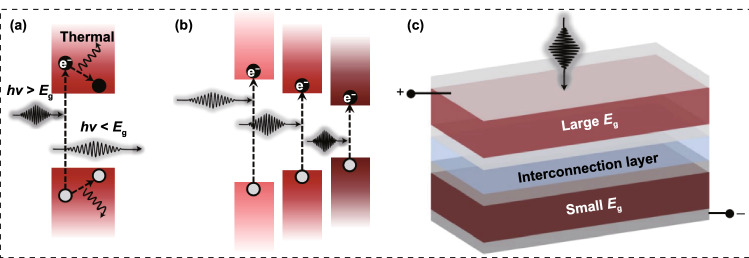
Schematic illustration of light absorption in **a** single and **b** multi-junction solar cells. **c** Two-terminal tandem devices. Reproduced with permission from Ref. [22]. Copyright 2021, Nature Publishing Group

In addition to fascinating optoelectronic properties like high light-absorption coefficient, bipolar carrier transport, high carrier mobility and low exciton binding energy, the tunable bandgap and solution processibility make perovskite materials desirable candidates for the preparation of TSCs [18–21]. Taking silicon solar cells as example, its efficiency improvement is significantly restricted by the thermalization loss [22]. That is, the photons at short wavelength possess extremely higher energy than the bandgap of Si absorber, leading to the excess energy loss via thermal relaxation of hot carriers as the excited electrons falling back to the band edge (Fig. 1a). In order to simultaneously absorb more photons and reduce thermalization loss (Fig. 1b), it is feasible to construct multi-junction solar cells via bandgap complementarity strategy. In a multi-junction solar cell, the front cell with WBG absorber captures high-energy photons to reduce the thermalization loss and deliver high open-circuit voltage (*V*_OC_), while the rear cell with NBG absorber harvests low-energy photons to broaden the photoresponse (Fig. 1c) [23–25]. Therefore, taking advantage of tunable bandgap (1.20–2.3 eV) of perovskite materials, different kinds of perovskite-based tandem solar cells can be obtained [26–28]. To date, there are mainly four types of perovskite-based monolithic TSCs: perovskite/silicon, perovskite/CIGS, all-perovskite and perovskite/organic, which deliver certified PCEs of 32.5%, 24.2%, 29% and 23.4%, respectively [1, 29–31]. Beyond monolithic two-terminal (2-T) TSCs, mechanically stacked four-terminal (4-T) TSCs have also been widely studied [11, 32]. In a 4-T architecture, the two sub-cells are fabricated independently and mechanically stacked. The independent processing of the sub-cells allows each sub-cell to work at their maximum power point conditions, which makes 4-T tandem cells less sensitive to spectral changes. In other words, for 4-T tandems, the demand for the bandgap of perovskites is not strict. The key point is developing highly efficient semitransparent PSCs. However, the 4-T tandems suffer from some inherent disadvantages such as additional balance-of-system (BOS) costs of separate system components and severe parasitic absorption losses due to the use of more transparent electrodes. While a 2-T architecture consists of two sub-cells connected in series through a charge recombination layer, which requires less transparent electrode, showing the advantages of reducing costs and parasitic absorption losses [33]. So far, the highest efficiencies for 2-T and 4-T TSCs have reached 32.5% [1] and 30.24% [34], respectively. The extremely higher PCE makes 2-T perovskite-based TSCs become the research frontier in photovoltaic field, showing great potential for commercialization. As the key front absorber in TSCs, WBG perovskites are vital for all 2-T perovskite-based TSCs. Highly efficient and stable WBG PSCs are important guarantee for high-performance TSCs. Hence, WBG PSCs have attracted increasing attention in recent years [35]. Reviewing the encouraging progress is meaningful to promote the further development of WBG PSCs and tandem application.

Herein, we reviewed recent progress of WBG PSCs from the aspects of compositions, additives, charge transport layers, interfaces and preparation methods, in which the key factors affecting device performance are carefully discussed. It is highlighted that challenges including *V*_OC_ deficit, stability and module fabrication are still the major concerns for WBG PSCs and monolithic tandem application. In the end, we gave an outlook on the strategies toward the highly efficient, long-term stable and large-area monolithic perovskite-based TSCs.

## Perovskite Materials

### Crystal Structure

**Fig. 2:**
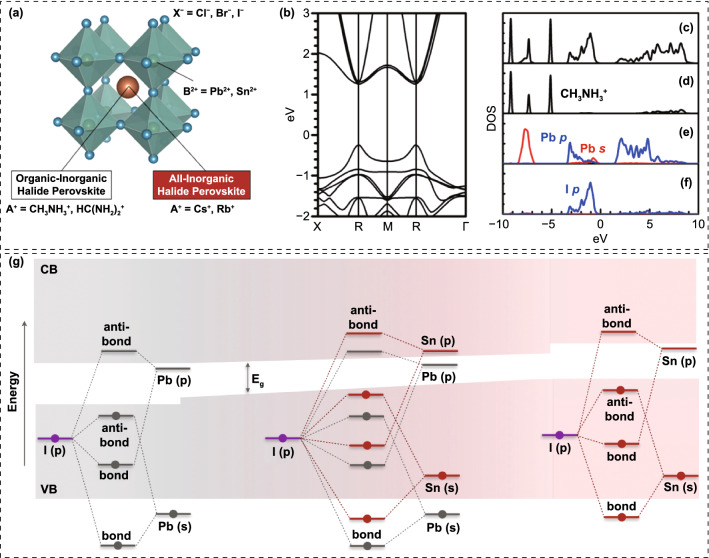
**a** Typical ABX_3_ crystal structure of halide perovskites. Reproduced with permission from Ref. [36]. Copyright 2019, Royal Society of Chemistry. **b** Band structure. **c** Total DOS. Partial DOSs of **d** MA^+^, **e** Pb, and **f** I. Reproduced with permission from Ref. [40]. Copyright 2014, AIP Publishing LLC. **g** Schematic of origin of the bandgap bowing in MA(Pb_1−x_Sn_x_)I_3_. Reproduced with permission from Ref. [44]. Copyright 2018, American Chemical Society

The organic–inorganic hybrid perovskite materials have a typical ABX_3_ perovskite structure (Fig. 2a) [36]. The A site is monovalent cation such as methylammonium (MA^+^), formamidinium (FA^+^) and Cs^+^, B site is divalent cation such as Pb^2+^ and Sn^2+^, and X site is halide ion such as I^−^, Br^−^ and Cl^−^. In the ABX_3_ perovskite structure, the A and B cations occupy the corner and center of the cubic unit cell, respectively, while the X anion locates at the face-center. The formability and stability of this structure is commonly evaluated by tolerance factor *t* and octahedral factor *μ*, defined as:

1 $$t=\frac{{r}_{\mathrm{A}}+{r}_{\mathrm{X}}}{\surd 2({r}_{\mathrm{B}}+{r}_{\mathrm{X}})}$$

2 $$\mu =\frac{{r}_{\mathrm{B}}}{{r}_{\mathrm{X}}}$$

where *r*_A_, *r*_B_ and *r*_X_ are the ionic radii of the A, B and X ions, respectively. Typically, when 0.8 < *t* < 1.0, the crystal shows a 3D perovskite structure, and a *μ* value between 0.4 and 0.9 favors a stable BX_6_ octahedra [37–39].

### Electronic Structure

The electronic structure of hybrid perovskite has been studied by density functional theory (DFT) calculations (Fig. 2b–f) [40]. It is revealed that the conduction band minimum (CBM) is dominated by the antibond from the Pb-*p* orbital, and the valence band maximum (VBM) is determined by the antibond from the Pb–*s* and I-*p* orbitals (Fig. 2c–f). Take MAPbI_3_ perovskite for example, the optical transition of MAPbI_3_ depends on a direct bandgap *p-p* transition, giving rise to high absorption coefficient. The extremely high absorption coefficient (10^4^~10^5^ cm^−1^) gifts them ability of absorbing sufficient sunlight with a thin film (< 1 μm). The well-dispersed bands near CBM and VBM (Fig. 2b) result in the small effective electron mass (*m*_e_^*^) and hole mass (*m*_h_^*^), which is responsible for the efficient bipolar carrier transport property of perovskite materials [41, 42].

### Tunable Bandgap

The bandgap of perovskite materials can be continuously adjusted via composition engineering. All the *A*, *B* and *X* sites have significant influences on the bandgap of perovskite materials. For instance, the bandgaps for MAPbI_3_, FAPbI_3_ and CsPbI_3_ perovskites are about 1.55, 1.48, and 1.73 eV, respectively. That is, the bandgap increases as the ionic radius of A-site decreases, which is due to the modulation of the Pb-I bond originating from the size effects of A-site cations [43]. Substituting Pb with Sn will result in a remarkable reduction in bandgap, making Pb–Sn mixed perovskites ideal rear sub-cells for all-perovskite TSCs (Fig. 2g) [44, 45]. *X*-site regulation is the most widely used approach for adjusting perovskite bandgap. As the X ions in MAPbX_3_ vary from I to Br to Cl, the bandgap will increase from 1.55 to 1.80 to 2.30 eV [46]. Particularly, as reported in previous literatures, the MAPbI_3-x_Cl_x_ perovskite shows similar bandgap with MAPbI_3_. The reason for the minor effect of Cl-doping could be the little amount of Cl into the lattice [47]. Interestingly, for Br-rich organic–inorganic hybrid perovskites or all-inorganic perovskites, the Cl is capable of occupying *X*-sites and further widening the bandgap, which is possibly due to that Cl can replace the Br but not I considering the difference in ion radius [48]. Generally, for hybrid perovskites with mixed halides and different A and B cations, the X site plays a more prominent impact on bandgap. For example, the WBG organic–inorganic hybrid perovskites are usually obtained by heavy Br doping. Certainly, if we fix the bandgap and reduce Br content, the Cs or MA content should be increased in CsMAFAPbI_3-x_Br_x_ systems. In addition to composition, the dimensionality of the crystal structure also dramatically influences the bandgap of perovskites [49]. It should be noted that the bandgap increases as the structural dimensionality decreases [50]. For example, in the quasi-two-dimensional (2D) perovskites, the bandgap increases as the *n* value decreases [51].

## WBG Perovskites for Tandem Application

### Current Matching for Monolithic TSCs

**Fig. 3:**
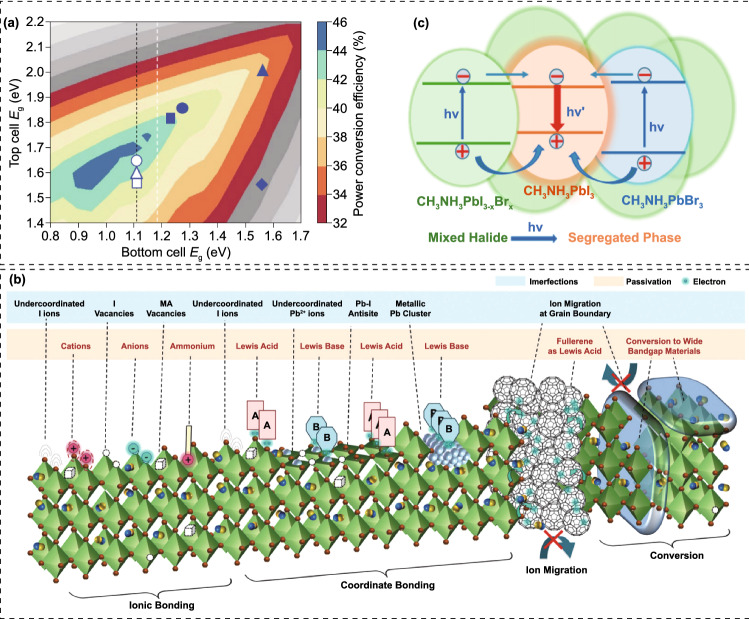
**a** Theoretical efficiency limit for 2-T tandems. Reproduced with permission from Ref. [55]. Copyright 2018, Nature Publishing Group. **b** Defects (imperfections) in perovskite film and their passivation strategies. Reproduced with permission from Ref. [61]. Copyright 2019, Royal Society of Chemistry. **c** Phase segregation and corresponding charge transfer behavior. Reproduced with permission from Ref. [62]. Copyright 2016, American Chemical Society

The monolithic or named two-terminal (2-T) perovskite-based TSC is consisted of a WBG PSC, a narrow-bandgap solar cell such as Si, a recombination layer, and a transparent electrode [52]. The WBG absorber harvests high-energy photons, and the transmitted low-energy photons are captured by NBG absorber. The opposite charge carriers produced by the two sub-cells arrive at the recombination layer, thus achieving monolithic circuit [53, 54]. The *V*_OC_ of the 2-T tandems is equal to the sum of the *V*_OC_ of the two sub-cells. Whereas, the short-circuit current (*J*_SC_) of a 2-T TSC is limited by the sub-cell with lowest *J*_SC_. Therefore, the current matching is extremely important for 2-T TSCs, which means that the bandgap and the thickness of the two absorbers require precise combination. For the above mentioned four types of 2-T perovskite-based TSCs, owing to the different bandgaps of NBG absorbers, the corresponding optimum bandgaps of WBG perovskites are also different (Fig. 3a) [55]. For Si and CIGS with similar bandgap of 1.10 eV, the required bandgap of front perovskite absorber is about 1.68 eV. For all-perovskite TSCs, the bandgaps of WBG and NBG perovskites are about 1.78 and 1.25 eV, respectively [56]. And for perovskite/organic TSCs, the absorption cut-off edge of organic heterojunction locates at 950 nm, and the matched bandgap of perovskite is about 1.80 eV [15]. Table 1 summarized the performance data of highly efficient perovskite-based TSCs.

**Table 1:** Summary of highly efficient perovskite-based TSCs

Top Cell	Bottom Cell	*V*_OC_ (V)	*J*_SC_ (mA cm^−2^)	FF (%)	PCE (%)	References
Cs_0.22_FA_0.78_PbI_2.55_Br_0.45_	Si	1.886	19.12	75.3	27.13	[99]
Cs_0.22_FA_0.78_Pb(I_0.85_Br_0.15_)_3_	Si	1.86	19.23	76.22	27.26	[100]
Cs_0.15_(FA_0.83_MA_0.17_)_0.85_Pb(I_0.8_Br_0.2_)_3_	Si	1.80	17.8	79.4	25.4	[141]
Cs_0.1_FA_0.2_MA_0.7_Pb(I_0.85_Br_0.15_)_3_	Si	1.92	18.95	78.5	28.56	[147]
(FA_0.65_MA_0.20_Cs_0.15_)Pb(I_0.8_Br_0.2_)_3_	Si	1.756	19.1	79.2	26.7	[149]
Cs_0.05_FA_0.8_MA_0.15_Pb(I_0.75_Br_0.25_)_3_	Si	1.870	19.6	78.6	28.9	[156]
Cs_0.22_FA_0.78_Pb(I_0.85_Br_0.15_)_3_	Si	1.185	18.04	80.76	26.95	[69]
Cs_0.05_FA_0.8_MA_0.15_Pb(I_0.75_Br_0.25_)_3_	Si	1.790	19.5	79.6	27.8	[177]
FA_0.75_Cs_0.25_Pb(I_0.8_Br_0.2_)_3_	Si	1.77	18.4	77	25.0	[178]
NA	Si	1.794	20.11	79.95	28.84	[188]
FA_0.8_Cs_0.2_PbI_1.8_Br_1.2_	Si	1.75	18.4	78.9	25.4	[191]
Cs_0.05_MA_0.15_FA_0.8_Pb(I_0.85_Br_0.15_)_3_	Si	1.828	19.5	75.9	27.1	[198]
Cs_0.15_FA_0.85_Pb(I_0.75_Br_0.27_)_3_	Si	1.808	19.78	76.85	27.48	[194]
Cs_0.15_MA_0.15_FA_0.7_Pb(I_0.8_Br_0.2_)_3_	Si	1.84	19.6	76.0	27.4	[224]
Cs_0.05_(FA_0.77_MA_0.23_)_0.95_Pb(I_0.77_Br_0.23_)_3_	Si	1.90	19.23	79.4	29.01	[107]
Cs_0.05_FA_0.8_MA_0.15_Pb(I_0.755_Br_0.255_)_3_	Si	1.92	19.8	80.7	30.5	[13]
FA_x_Cs_1-x_Pb(I_y_Br_1-y_)_3_	Si	1.83	18.99	79.46	27.64	[238]
Cs_x_FA_1−x_Pb(I,Br)_3_	Si	1.788	19.53	73.1	25.52	[193]
Cs_0.05_MA_0.15_FA_0.8_PbI_2.25_Br_0.75_	Si	1.70	19.8	77	26.0	[243]
Cs_0.1_MA_0.9_Pb(I_0.9_Br_0.1_)_3_	Si	1.82	19.2	75.3	26.2	[180]
DMA_0.1_FA_0.6_Cs_0.3_PbI_2.4_Br_0.6_	PVK	1.88	16.0	77	23.1	[82]
DMA_0.1_Cs_0.4_Br_0.25_Cl_0.05_	PVK	2.046	16.0	80.1	26.2	[72]
FA_0.8_Cs_0.2_PbI_1.8_Br_1.2_	PVK	2.0	15.6	79.9	24.9	[191]
Cs_0.4_FA_0.6_PbI_1.95_Br_1.05_	PVK	2.03	15.2	79.7	24.6	[199]
Cs_0.35_FA_0.65_PbI_1.8_Br_1.2_	PVK	2.025	15.4	79.4	24.8	[110]
Cs_0.05_(MA_0.17_FA_0.83_)_0.95_Pb(I_0.83_Br_0.17_)_3_	GIGS	1.68	19.17	71.9	23.16	[183]
Cs_0.05_(MA_0.23_FA_0.77_)Pb_1.1_(I_0.77_Br_0.23_)_3_	GIGS	1.77	18.8	71.2	23.7	[29]
Cs_0.09_FA_0.77_MA_0.14_Pb(I_0.86_Br_0.14_)_3_	GIGS	1.774	17.3	73.1	22.43	[9]
MA_0.96_FA_0.1_PbI_2_Br(SCN)_0.12_	OPV	1.96	13.8	78.4	21.2	[80]
CsPbI_2_Br	OPV	2.22	12.68	76.0	21.4	[119]
CsPbI_1.8_Br_1.2_	OPV	2.05	13.36	76.82	21.04	[120]
FA_0.6_MA_0.4_Pb(I_0.6_Br_0.4_)_3_	OPV	1.88	15.7	74.6	22.0	[148]

### *V*_OC_ Deficit

In contrast to the normal bandgap PSCs, WBG PSCs suffer from more serious *V*_OC_ loss, which significantly affect the efficiency improvement [57]. As is known to all, the *V*_OC_ is determined by the quasi-Fermi level (*E*_F_) splitting of the perovskite under illumination [58]. The WBG perovskites are expected to offer higher *V*_OC_. For WBG perovskites, the CBM and VBM are up and down shifted in some degree, respectively. When contacts with charge transport layers, the quasi-*E*_F_ of WBG perovskite is counterbalanced. The relatively larger energy gap between perovskite and charge transport layers (CTLs) would arise energy loss and interfacial non-radiative charge recombination [59]. Therefore, desirable energy level matching between perovskite and CTLs is essential for higher *V*_OC_. Moreover, the trap-assisted non-radiative recombination is recognized as the major killer for *V*_OC_ [60]. Deep-level trap states in the forbidden band will retard quasi-*E*_F_ splitting and suppress initial radiative efficiency, thus dramatically limits the *V*_OC_ improvement. Since grain boundaries and film surface accommodate amounts of trap states during the film formation, passivating these defects is preferred to suppress non-radiative recombination and reduce *V*_OC_ loss (Fig. 3b) [61].

### Photo-induced Phase Segregation

For most WBG perovskites in TSCs, they are Br-rich organic–inorganic hybrid perovskites, which undergo phase separation and arise lower bandgap I-rich and higher-bandgap Br-rich domains under continuous illumination (Fig. 3c) [62–65]. Upon the formation of I-rich region, they would act as the carrier recombination centers irrespective of the carrier generation position in the mixed halide perovskites. This is in consistent with the results of photoluminescence (PL) measurements, which reveals the red shift of PL emission peaks after light-soaking [66, 67]. In other words, the generated charge carriers would quickly thermalize and accumulate upon encountering any I-rich region. The difference in band structure between uniformly mixed perovskite and I-rich region could also create an electric field that might further help to sweep more carriers into the I-rich region. Hence, nearly all of the PL emission of light-soaked perovskites should come from the radiative relaxation of carriers trapped in I-rich regions. It is believed that the phase segregation is originated from halide migration via halide vacancy, which is more facile at grain boundaries [68–70]. Some results also suggested employing FA or Cs instead of MA as the A-site cation can significantly enhance the photostability, and improving the spatial homogeneity to release lattice microstrain and increase kinetic barrier for ion migration might eliminate or slow phase segregation [71]. Therefore, optimizing the composition and lattice structure, improving crystallinity and compositional uniformity, and reducing trap state density could significantly improve the photostability.

### Crystallization Control

It is well known that the quality of perovskite films directly determines the efficiency and stability of the PSCs. Different from normal-bandgap perovskites, the solution-processed Br-rich WBG perovskites undergo faster crystallization, which would result in inferior crystallinity, more trap states and irreversible microstrain [72]. The trap-states would act as non-radiative recombination centers to impede the improvement of device performance [60, 61]. Modulating the perovskite crystal growth and suppressing the formation of defects are the major approaches to obtain high-quality WBG perovskite films [73]. Interface engineering and additive engineering are promising strategies to modulate crystallization and passivate defects.

### Long-Term Stability

The major challenge for the commercialization of WBG PSCs and TSCs is the inferior long-term operation stability compared to commercial silicon solar cells [74]. The factors influencing the device stability could be divided into intrinsic phase stability issues and external factors such as UV light, oxygen, moisture, temperature [75, 76]. The organic–inorganic halide perovskites perform poor thermal, air, and light stabilities due to the ionic crystal nature [77]. High-quality perovskite film is fundamentally important for long-term stability. It has been demonstrated that strategies such as compositional engineering, structural engineering and additive engineering can significantly enhance the stability of the PSCs. In addition, the instability of charge transport layers (CTLs) is another key degradation pathway for WBG PSCs [78]. Chemically stable CTLs are also crucial for improving the long-term stability of WBG PSCs and TSCs.

## Optimization for WBG Perovskites

### Composition Engineering

The composition of WBG perovskites plays a critical role in the device performance because various perovskites in spite of similar bandgap might present different crystal and electronic structures, which might influence the formation of trap states in the perovskite films [79–82]. These trap states are directly responsible for the non-radiative recombination and instability issues. Therefore, it is of great significance to investigate the deep relationship between composition and device performance of WBG PSCs, which is fundamentally important for providing more efficient and pointed guidance for the further optimization [83–85].

**Fig. 4:**
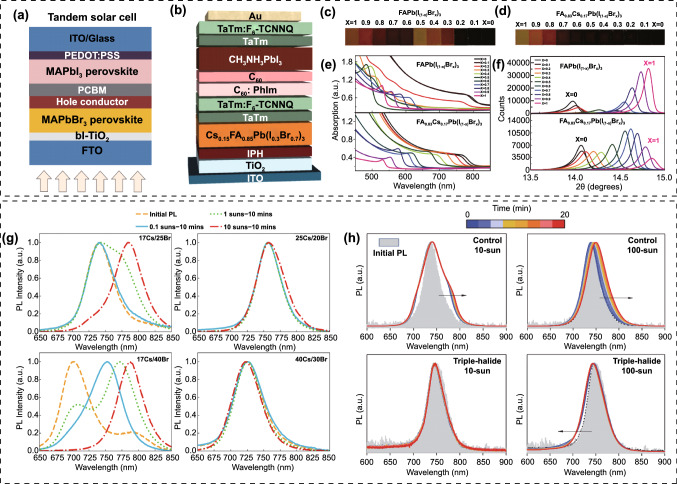
**a** Device structure of MAPbBr_3_-MAPbI_3_ 2-T tandem cell. Reproduced with permission from Ref. [86]. Copyright 2016, Wiley–VCH. **b** Device structure of Cs_0.15_FA_0.85_Pb(I_0.3_Br_0.7_)_3_-MAPbI_3_ 2-T tandem cell. Reproduced with permission from Ref. [93]. Copyright 2017, Wiley–VCH. Photographs of perovskite films with Br composition increasing from x = 0 to 1 for **c** FAPb[I_(1-x)_Br_x_]_3_ and **d** FA_0.83_Cs_0.17_Pb[I_(1-x)_Br_x_]_3_. **e** Uv–vis absorption spectra of films of FAPb[I_(1-x)_Br_x_]_3_ and FA_0.83_Cs_0.17_Pb[I_(1-x)_Br_x_]_3_. **f** XRD pattern of FAPb[I_(1-x)_Br_x_]_3_ and FA_0.83_Cs_0.17_Pb[I_(1-x)_Br_x_]_3_. Reproduced with permission from Ref. [96]. Copyright 2015, Science Publishing Group. **g** PL spectra of Cs17/Br25, Cs25/Br20, Cs17/Br40, Cs40/Br30 perovskites. The initial PL peak is shown as the orange dashed curve. Reproduced with permission from Ref. [98]. Copyright 2018, American Chemical Society. **h** PL spectra of control perovskite films (Cs25Br20) and triple-halide perovskites (Cs22Br15 + Cl3) under 10-sun and 100-sun illumination for 20 min, respectively. Reproduced with permission from Ref. [99]. Copyright 2020, Science Publishing Group

The early research on WBG perovskites mainly focused on MAPbI_3-x_Br_x_. In 2015, Heo et al*.* reported a 2-T all-perovskite TSC employing a MAPbBr_3_ front cell and a MAPbI_3_ rear cell, which delivered a PCE of 10.4% with a high *V*_OC_ of 2.25 V (Fig. 4a) [86]. Huang’s group tuned the bandgap of MAPbI_3-x_Br_x_ to 1.72 eV via two-step preparation method, in which the MABr and MAI mixed isopropanol solution with different blend ratios were spin-coated onto the PbI_2_ layer, followed by solvent annealing process [87]. And the target MAPbI_2.4_Br_0.6_ solar cell demonstrated a PCE of 13.1%. Zhu et al*.* prepared a 1.75 eV bandgap MAPbI_2.1_Br_0.9_ perovskite film via a facile halide exchange route, that is, a parent MAPbI_3_ film was firstly deposited, and then dipped into MABr isopropanol solution for halide exchange with different reaction time [88]. This approach contributes to more dense and homogenous film with vertically oriented crystals as compared with one-step preparation method, and a 12.67% efficiency was obtained. Since the (FAPbI_3_)_0.85_(MAPbBr_3_)_0.15_ was firstly reported, the FA-based perovskites gradually replaced the MA system as the focus due to the higher efficiency and better stability [89]. And inorganic Cs and Rb cations were also incorporated to further modulate the crystallization and improve the device performance [90–92]. Forgács et al*.* presented a 2-T all-perovskite TSC by using 2.0 eV bandgap Cs_0.15_FA_0.85_Pb(I_0.3_Br_0.7_)_3_ as the front absorber, which delivered PCEs of 10.7% and 15.6% for single-junction and tandem solar cells, respectively (Fig. 4b) [93, 94]. Later, they further improved the PCE of Cs_0.15_FA_0.85_Pb(I_0.3_Br_0.7_)_3_ solar cells up to 11.5% [95]. McMeekin et al*.* found that partially substituting the FA with Cs can eliminate the phase instability region in the I-to-Br compositional range, over which the variation in composition, lattice constant, and bandgap precisely follows Vegard's law (Fig. 4c-f) [96]. The Cs_0.17_FA_0.83_Pb(I_0.6_Br_0.4_)_3_ perovskite exhibited a 1.74 eV bandgap and gave a PCE of 17.1% with 1.2 V *V*_OC_, as a result of the suppressed phase separation. Yang et al*.* demonstrated that the use of non-stoichiometric precursor with excess MABr or MACl can significantly improve the crystallinity of Cs_0.17_FA_0.83_Pb(I_0.6_Br_0.4_)_3_ perovskite but without affecting the bandgap [97]. In contrast, an obvious reduction in bandgap was observed when excess MAI was added, which was resulted from the substitution of Br with I. Consequently, the MABr incorporated Cs_0.17_FA_0.83_Pb(I_0.6_Br_0.4_)_3_ solar cell delivered a highest stabilized PCE of 16.4%.

As either the incorporation of Cs or Br can enlarge the bandgap, there should be a ideal Cs/Br ratio to simultaneously improve *V*_OC_ and photostability. In view of this, Bush et al*.* systematically investigated the device performance of Cs_x_FA_1-x_Pb(Br_y_I_1-y_)_3_ with various A and X sites compositions [98]. It was revealed that Cs17/Br25 and Cs25/Br20 perovskites with bandgap of 1.68 eV demonstrate different performance, so as the Cs17/Br40 and Cs40/Br30 with bandgap of 1.75 eV. For the two conditions, the slightly high Cs and low Br gifts higher *V*_OC_. Moreover, the PL results showed that high Cs and low Br results in better photostability, while low Cs and high Br leads to remarkable halide segregation (Fig. 4g). For the target bandgaps of 1.68 eV and 1.75 eV for tandem application, the Cs25/Br20 and Cs40/Br30 afforded higher PCEs of 17.4% and 16.3%, respectively, and significantly improved photostability. In order to further decrease the Br content and retain desirable bandgap for perovskite/silicon tandems, Xu et al*.* proposed a triple-halide alloys (I, Br, Cl) method to tailor the bandgap via introducing additional 3 mol% MAPbCl_3_ into Cs_0.22_FA_0.78_Pb(I_0.85_Br_0.15_)_3_ perovskite [99]. The resulted perovskite film shows a enlarged bandgap from 1.63 to 1.67 eV, increased carrier lifetime and mobility, and suppressed light-induced phase segregation (Fig. 4h). As a result, the single-junction and perovskite/silicon solar cells offered PCEs of 20.42% and 27%, respectively. Based on this recipe, Li et al*.* added 3 mol% inorganic CsPbCl_3_-clusters instead of MAPbCl_3_ into the perovskite precursor, and obtained a 1.67 eV bandgap as well [100]. Moreover, they further added extra 2 mol% CsCl to enrich the NiO/perovskite interface with Cl, thus suppressing the redox reaction at the interface. The reduction in interfacial non-radiative recombination effectively improves *V*_OC_. Consequently, the PCE of single-junction solar cell improves from 17.82% to 19.76%, giving rise to a 27.26% efficiency for perovskite/silicon tandem cell. Besides, it can be concluded that PbCl_2_ but not MACl and CsCl plays a critical role in tailoring the bandgap of Br-rich WBG perovskites.

**Fig. 5:**
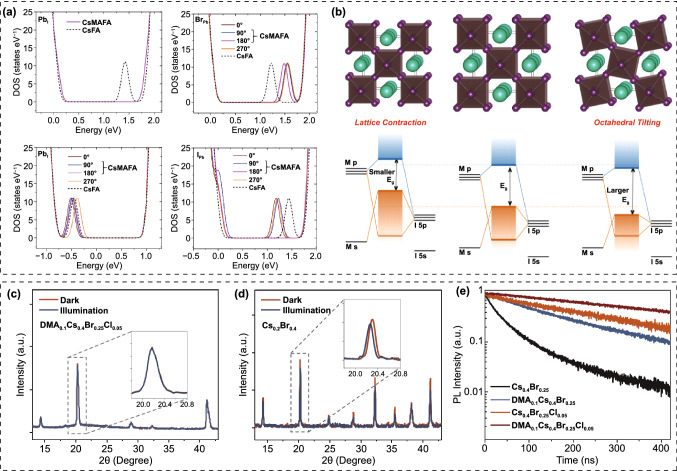
**a** Density of states (DOS) of CsFA and CsMAFA perovskites in the case of anti-site defects. Reproduced with permission from Ref. [106]. Copyright 2018, Nature Publishing Group. **b** Perovskite lattice diagrams: undistorted cubic (center panel), with lattice contraction (left panel) and with octahedral tilting (right panel). Below each lattice is a schematic energy level diagram, showing how each kind of distortion affects the valence and conduction bands. Reproduced with permission from Ref. [108]. Copyright 2017, American Chemical Society. XRD patterns and magnified (110) diffraction peaks (inset) of **c** DMA_0.1_Cs_0.4_Br_0.25_Cl_0.05_ and **d** Cs_0.2_Br_0.4_ perovskite films under dark and 1 sun illumination. **e** TRPL spectra of Cs_0.4_Br_0.25_, DMA_0.1_Cs_0.4_Br_0.25_, Cs_0.4_Br_0.25_Cl_0.05_, and DMA_0.1_Cs_0.4_Br_0.25_Cl_0.05_ perovskite films on glass substrates. Reproduced with permission from Ref. [72]. Copyright 2022, Wiley–VCH

In addition to Cs_x_FA_1-x_PbI_3-y_Br_y_ WBG systems, mixed A-sites CsMAFA compositions were also widely investigated. Zhou and co-workers prepared four WBG perovskites with different bandgaps, that are FA_0.48_MA_0.37_Cs_0.15_PbI_2.23_Br_0.77_ (1.65 eV), FA_0.57_MA_0.43_PbI_2.4_Br_0.96_ (1.69 eV), FA_0.5_MA_0.38_Cs_0.12_PbI_2.04_Br_0.96_ (1.69 eV) and FA_0.51_MA_0.38_Cs_0.11_PbI_1.85_Br_1.15_ (1.72 eV) for 2-T perovskite/silicon TSCs, wherein the perovskite with 1.69 eV bandgap delivered the best efficiency [101]. Though the FA_0.57_MA_0.43_PbI_2.4_Br_0.96_ and FA_0.5_MA_0.38_Cs_0.12_PbI_2.04_Br_0.96_ have similar bandgap, the Cs-contained system exhibits much higher carrier lifetime than that of composition without Cs, highlighting the effectiveness of triple A-site strategy for high-performance PSCs. Chen et al*.* reported four WBG perovskites with different A-site combinations and fixed Br content, that are Cs_0.6_MA_0.4_Pb(I_0.6_Br_0.4_)_3_, Cs_0.6_FA_0.4_Pb(I_0.6_Br_0.4_)_3_, MA_0.6_FA_0.4_Pb(I_0.6_Br_0.4_)_3_ and FA_0.6_MA_0.4_Pb(I_0.6_Br_0.4_)_3_ [102]. Interestingly, they found that the Cs-MA and Cs-FA combinations demonstrate better photostability than that of MA-FA combinations, but the MA-FA device delivered the highest *V*_OC_ and PCE. It has been proposed that the collective rotation of dipolar MA^+^ in MAPbX_3_ perovskites can enhance charge transport owing to the formation of large polarons or the spatially localized carriers [103]. The liquid-like reorientation and dipolar disorder of MA^+^ can protect hot carriers and increase the dielectric constants for MAPbX_3_ perovskites [86] 104. Moreover, calculations also revealed that the deep traps in MAPbI_3_ can be healed by dynamic rotation of MA^+^ [105]. Inspired by these studies, Tan et al*.* demonstrated that the higher density of deep traps in the WBG perovskites than that of sub-1.6 eV bandgap PSCs causes the inferior performance, and they proposed that increasing the defect tolerance via A-site engineering can heal the deep traps and enable further performance improvement for WBG PSCs [106]. They elucidated that the reorientation of dipolar MA^+^ can eliminate or render innocuously shallow electronic states, which could evolve into deep traps if the MA is absent (Fig. 5a). Consequently, the Cs_0.2_FA_0.8_Pb(I_0.75_Br_0.25_)_3_ (1.67 eV), Cs_0.05_MA_0.15_FA_0.8_Pb(I_0.75_Br_0.25_)_3_ (1.65 eV), Cs_0.17_FA_0.83_Pb(I_0.6_Br_0.4_)_3_ (1.74 eV) and Cs_0.12_MA_0.05_FA_0.83_Pb(I_0.6_Br_0.4_)_3_ (1.74 eV) solar cells offered PCEs of 18.5%, 20.8%, 17.2% and 19.3%, respectively. Carefully comparing the device performance of WBG PSCs with various compositions (Table 2), we can found that despite of similar bandgap, the CsMAFA system performs better than CsFA in respect of efficiency. For more examples, Al-Ashouri et al*.* employed Cs_0.05_(FA_0.77_MA_0.23_)_0.95_Pb(I_0.77_Br_0.23_)_3_ perovskite as the front absorber, the single-junction solar cell gave a PCE of 20.8%, and the 2-T perovskite/silicon tandem solar cell achieved an impressive PCE of 29.15% [107]. Also, Liu et al*.* adopted Cs_0.05_FA_0.8_MA_0.15_Pb(I_0.75_Br_0.25_)_3_ perovskite as the front cell, which offered 20.46% and 29.3% efficiencies for single-junction and tandem solar cells, respectively [13]. The detailed discussion of these two works will be introduced in the following part of “*Interface engineering*”. This interesting phenomenon deserves deep excavation. For CsFA systems, in order to avoid too much Br content that account for photo-instability, heavy Cs-doping (> 15%) is commonly required for the desirable wide bandgap. As thus, there is a innegligible size mismatching in ionic radius, 1.88 Å for Cs^+^ and 2.53 Å for FA^+^, which would induce PbX_6_ octahedra tilting and lattice microstrain (Fig. 5b) [108]. For instance, both of FA_0.85_Cs_0.15_PbI_3_ and FA_0.75_Cs_0.25_PbI_3_ are assigned to tetragonal space group P4/mbm, indicating a reduction in the metal-halide-metal bond angle below 180° that for a cubic structure. Considering the σ* interaction in the metal-halide orbital overlap, the reduction in the bond angle will reduce the degree of overlap between metal and halide orbitals, pushing the valence band toward lower energy and increasing the bandgap as the Cs substitution increases. Whereas, for CsMAFA systems, the relatively lower Cs content leads to less octahedra tilting. Moreover, the MA^+^ (2.17 Å) would act as buffer cations to minimize the size mismatch between Cs^+^ and FA^+^ and thus, further suppress the octahedra tilting. The larger octahedra tilting in the heavy Cs-doped CsFA system than that of CsMAFA might introduce additional trap states and thus, sacrifice the *V*_OC_ and PCE. Whereas, Cs-rich and low-Br WBG perovskites deserve further exploration because of their great potential for highly efficient and photostable PSCs. Therefore, choosing suitable A-site cations to eliminate size mismatch is a promising strategy for Cs-rich PSCs. In addition to MA^+^, DMA^+^, EA^+^ and Gua^+^, etc*.*, were also reported to improve the structural stability [82, 109]. Palmstrom and co-workers incorporated 10% DMA into CsFA perovskite to enable a 1.70 eV bandgap, and the Cs_0.3_FA_0.6_DMA_0.1_PbI_2.4_Br_0.6_ solar cell delivered a PCE of 19.6% [82]. Wen et al*.* reported 1.70 eV bandgap PSC with a composition of Cs_0.3_FA_0.6_DMA_0.05_Gua_0.05_I_2.4_Br_0.6_, which gave a PCE of 20.1% with high *V*_OC_ of 1.226 V. Tan’ group proposed a steric engineering strategy via introducing 10 mol% DMA and 5 mol% Cl into Cs_0.4_FA_0.6_Pb(I_0.75_Br_0.25_)_3_, to simultaneously realize bandgap tuning and strain relaxation [72]. The resulted film shows a 1.80 eV bandgap with only 25% Br. The co-incorporation of DMA and Cl can significantly promote the strain relaxation, thus suppressing the light-induced halide segregation (Fig. 5c, d) and improving the carrier lifetime (Fig. 5e). The WBG single-junction cell afforded a PCE of 17.7% with a high *V*_OC_ of 1.26 V, giving rise to a stabilized PCE of 26.0% for all-perovskite TSCs. Moreover, this group systematically tuned the Cs ratio of a MA-free 1.8 eV perovskite, in which the homogeneity of crystallization was effectively improved for the blade-coated Cs_0.35_FA_0.65_PbI_1.8_Br_1.2_ films over large area (20 cm^2^) [110]. And the all-perovskite tandem solar modules gave a high PCE of 21.7%. The mixed-halide perovskites suffer from the severe phase segregation, leading to I-rich and Br-rich phases under continuous illumination. Ji et al*.* reported pure-iodide WBG perovskites which can fundamentally solve the photo-instability issue [111]. Through further controlling precipitation kinetics of the crystallization process, the Cs_0.3_DMA_0.2_MA_0.5_PbI_3_ perovskite/silicon TSC gave a PCE of 29.4% with excellent photostability.

**Table 2:** Summary of WBG organic–inorganic hybrid PSCs employing composition engineering

Perovskite	*E*_g_ (eV)	*V*_OC_ (V)	*J*_SC_ (mA cm^−2^)	FF (%)	PCE (%)	References
Rb_0.05_(Cs_0.05_MA_0.17_FA_0.83_)_0.95_Pb(I_0.83_Br_0.17_)_3_	1.63	1.186	22.5	77	20.6	[90]
Cs_0.05_MA_0.15_FA_0.8_Pb(I_0.75_Br_0.25_)_3_	1.65	1.22	21.2	80.5	20.8	[106]
(FAPbI_3_)_0.85_(MAPbBr_3_)_0.2_	1.67	1.14	21.15	77.49	18.68	[79]
Cs_0.22_FA_0.78_PbI_2.55_Br_0.45_	1.67	1.217	20.18	83.16	20.42	[99]
Cs_0.22_FA_0.78_Pb(I_0.85_Br_0.15_)_3_	1.67	1.19	20.33	81.7	19.76	[100]
Cs_0.2_FA_0.8_Pb(I_0.75_Br_0.25_)_3_	1.67	1.17	20.4	77.3	18.5	[106]
Cs_0.25_FA_0.75_Pb(I_0.8_Br_0.2_)_3_	1.68	1.10	19.4	81	17.4	[98]
Cs_0.05_(FA_0.77_MA_0.23_)_0.95_Pb(I_0.77_Br_0.23_)_3_	1.68	1.224	20.7	82.0	20.8	[107]
Cs_0.05_FA_0.8_MA_0.15_Pb(I_0.755_Br_0.255_)_3_	1.69	1.226	20.58	81.1	20.46	[13]
DMA_0.1_FA_0.6_Cs_0.3_PbI_2.4_Br_0.6_	1.70	1.20	19.6	82	19.2	[82]
MA_0.96_FA_0.1_PbI_2_Br(SCN)_0.12_	1.72	1.19	18.65	78.4	17.4	[80]
MAPbI_2.4_Br_0.6_	1.72	1.02	17.5	73.7	13.1	[87]
FA_0.83_Cs_0.17_Pb(I_0.6_Br_0.4_)_3_	1.74	1.2	19.4	75.1	17.1	[96]
Cs_0.17_FA_0.83_Pb(I_0.6_Br_0.4_)_3_	1.74	1.22	18.7	75.6	17.2	[106]
Cs_0.12_MA_0.05_FA_0.83_Pb(I_0.6_Br_0.4_)_3_	1.74	1.25	19.0	81.5	19.3	[106]
MAPbI_2.1_Br_0.9_	1.75	1.01	18.19	69	12.67	[88]
FA_0.83_Cs_0.17_Pb(I_0.6_Br_0.4_)_3_	1.75	NA	17.97	NA	16.77	[97]
Cs_0.4_FA_0.6_Pb(I_0.7_Br_0.3_)_3_	1.75	1.17	17.5	80	16.3	[98]
MA_0.6_FA_0.4_Pb(I_0.6_Br_0.4_)_3_	1.75	1.17	16.0	78.6	14.7	[102]
Cs_0.6_MA_0.4_Pb(I_0.6_Br_0.4_)_3_	1.75	1.17	13.9	68.3	11.1	[102]
Cs_0.6_FA_0.4_Pb(I_0.6_Br_0.4_)_3_	1.75	1.12	14.0	77.4	12.1	[102]
(FA_0.58_GA_0.10_Cs_0.32_)Pb(I_0.73_Br_0.27_)_3_	1.75	1.22	16.3	73.2	14.6	[109]
DMA_0.1_Cs_0.4_Br_0.25_Cl_0.05_	1.8	1.263	17.4	79.7	17.7	[72]
MA_0.9_FA_0.1_Pb(I_0.6_Br_0.4_)_3_	1.81	1.21	17.8	79.5	17.1	[85]
Cs_0.15_FA_0.85_Pb(I_0.3_Br_0.7_)_3_	2	1.215	11.41	81.2	10.7	[93]
Cs_0.15_FA_0.85_Pb(I_0.3_Br_0.7_)_3_	2	1.18	12.32	79.0	11.5	[95]
MAPbIBr_2_	2.05	1.08	15.3	64.7	10.7	[94]
MAPbBr_3_	2.25	1.08	8.6	78	7.2	[86]

**Fig. 6:**
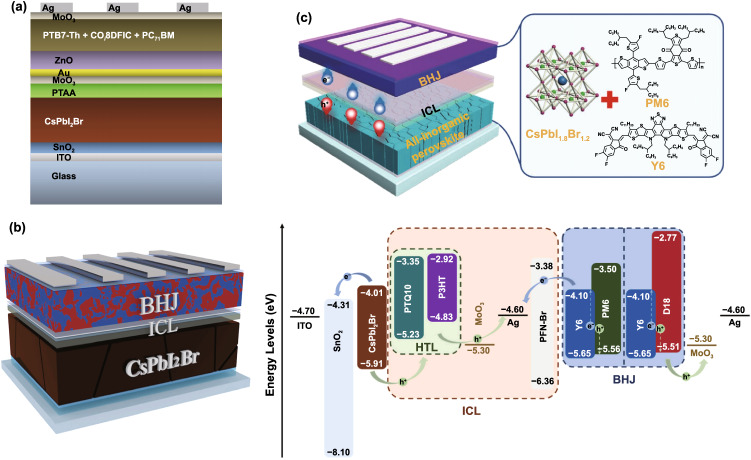
**a** Schematic for 2-T perovskite/organic TSC. Reproduced with permission from Ref. [117]. Copyright 2019, Elsevier. **b** Device structure and energy levels diagram of CsPbI_2_Br/organic TSC. Reproduced with permission from Ref. [119]. Copyright 2022, Elsevier. **c** Left: device structure of all-inorganic perovskite/organic TSC. Right: crystal structure of CsPbI_1.8_Br_1.2_ perovskite and the molecular structures of the donor and acceptor materials. Reproduced with permission from Ref. [120]. Copyright 2022, Wiley–VCH

Except from organic–inorganic halide perovskites, all-inorganic perovskites like CsPbI_3_, CsPbI_2_Br and CsPbBr_3_ have also attracted extensive attention due to their excellent thermal stability and the potential for tandem applications [112–116]. The bandgap of CsPbI_3_ perovskite ranges from 1.68 to 1.73 eV depending on the degree of PbI_6_ octahedral tilting. The desirable bandgap without Br incorporation makes CsPbI_3_ a promising candidate for perovskite/silicon tandems. Whereas, the major challenge for CsPbI_3-x_Br_x_ PSCs is the fraught phase stability against moisture due to its relatively low structural tolerance factor. In addition, preparing stable α-phase all-inorganic perovskites commonly requires high annealing temperature (> 200 °C), which might damage the underlying silicon cells. Also, for the sake of ideal light management, perovskite-based 2-T TSCs prefer *p-i-n* inverted PSCs. Because the hole transport layers like Spiro-OMeTAD and PTAA for the *n-i-p* structure is quite thick (~ 200 nm), which would arise additional parasitic absorption. The efficiency of all-inorganic PSCs with *p-i-n* structure lags behind that of *n-i-p* structure. Possibly considering the above concerns, there is still no report about CsPbI_3-x_Br_x_ based perovskite/silicon or CIGS, or all-perovskite tandems, which need be further exploited. Particularly, CsPbI_3-x_Br_x_/organic TSCs have been developed to improve the efficiency, and the CsPbI_3-x_Br_x_ front cell can harvest short-wavelength photons that might induce instability of organic solar cells. Ding’ group firstly demonstrated a CsPbI_2_Br/PTB7-Th:COi8DFIC:PC_71_BM tandem solar cells and delivered a PCE of 15.04% (Fig. 6a) [117]. Later, they further achieved a higher PCE of 20.18% with 2.05 V *V*_OC_ based on CsPbI_2_Br perovskite and D18:Y6 organic solar cell [118]. To date, the highest efficiency CsPbI_2_Br-based perovskite/organic TSCs was reported by Zhou’ group, which presented a PCE of 21.4% with an extremely high *V*_OC_ of 2.22 V (Fig. 6b) [119]. Besides, CsPbI_1.8_Br_1.2_ has also been employed for perovskite/organic TSCs, and a PCE of 21.04% was obtained (Fig. 6c) [120].

### Additive Engineering

Additive engineering has been widely used to modulate crystallization and passivate defects via various chemical interactions to improve the efficiency and stability of PSCs [121–124]. For WBG perovskites, the heavy Cs or Br contents would dramatically accelerate the crystallization process, resulting in inferior crystallinity, rough surface and more trap states, which can significantly limit the *V*_OC_ improvement and photostability of the devices [125]. Therefore, simultaneously optimizing the crystallization and passivating defects of WBG perovskite films via additive engineering is of great significance for high-performance PSCs.

**Fig. 7:**
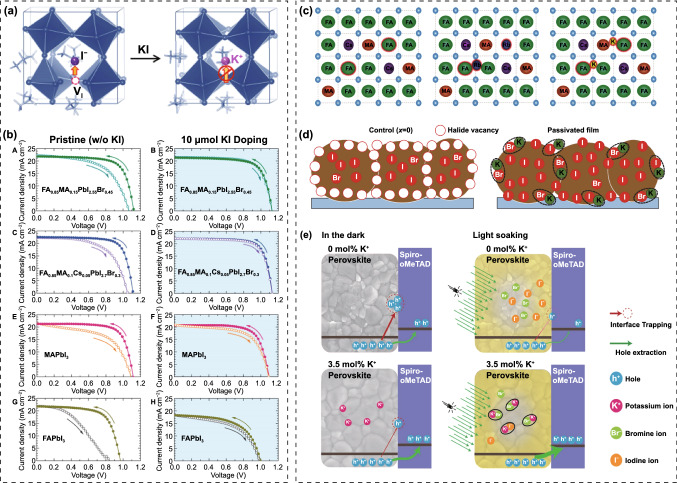
**a** Schematic of the Frenkel defect passivated by K^+^. **b** Current–voltage curves of PSCs employing different perovskite materials with and without K^+^ doping. Reproduced with permission from Ref. [128]. Copyright 2018, American Chemical Society. **c** Schematics of the doping mechanism of Cs/MA/FA perovskite doped with Cs^+^, Rb^+^, and K^+^. Reproduced with permission from Ref. [129]. Copyright 2018, Wiley–VCH. **d** Schematic of the passivation mechanism of K^+^, in which the surplus halide is immobilized through complexing with potassium into benign compounds at the grain boundaries and surfaces. Reproduced with permission from Ref. [130]. Copyright 2018, Nature Publishing Group. **e** Schematic of the passivation effect of K^+^-doped perovskite under illumination. Reproduced with permission from Ref. [132]. Copyright 2019, Wiley–VCH

Potassium (K^+^) ion doping has been identified as an effective approach to suppress *J-V* hysteresis and improve photostability. Tang et al*.* firstly explored the influence of K^+^ incorporation on the crystal structure and optical properties of perovskite films [126]. They observed the elongation of crystal lattice after K^+^ doping, which is opposite to the perovskites containing Cs^+^ and Rb^+^. Meanwhile, the absorption edge of the K^+^-doped film presented a red shift, indicating the decrease in bandgap. Most importantly, the conduction band minimum (CBM) matched well with the TiO_2_ layer, which is conducive to charge transfer, thus diminishing the *J-V* hysteresis. They hold the opinion that low concentration K^+^ was homogeneously incorporated into the crystal structure. Later, K^+^ attracted extensive interests for improving device performance of PSCs. Zhong and co-workers demonstrated that the hysteresis in PSCs can be completely removed by K^+^ irrespective of the electron transport layers [127]. The K^+^ can intensively improve the crystallization with grain size up to 1 μm, reduce the defect density, prolong the carrier lifetime and promote charge transport, contributing to the hysteresis-free stable and high efficiency PSCs. Son et al*.* proposed that the atomistic origin of the hysteresis of PSCs is the formation of iodide Frenkel defect but not the migration of iodide vacancy, and K^+^ is capable of inhibiting the formation of Frenkel defect because K^+^ energetically prefers the interstitial sites (Fig. 7a) [128]. Moreover, the suppression of hysteresis is more pronounced for mixed perovskites than that of pure perovskites due to the lower formation energy of K^+^ interstitial (−1.17 V for mixed perovskite and −0.65 V for MAPbI_3_) (Fig. 7b). Combining experimental and theoretical investigation, Cao et al*.* reported the size-dependent interstitial occupancy of extrinsic alkali cations (Li^+^, Na^+^, K^+^ and Rb^+^) incorporated into the CsMAFA perovskite [129]. Since these alkali cations are unfavorable for ideal Goldschmidt tolerance factor when occupy the A site of the Pb-I network due to small ionic radius, they attributed the lattice expansion to the interstitial occupancy by the alkali cations, which was confirmed by DFT calculations that interstitial occupancy is energetically more favorable than A site substitution (Fig. 7c). Furthermore, the calculations and characterization corroborated that the increased ion migration barriers after the interstitial occupancy account for the suppression of ion migration, and thus, the elimination of hysteresis. Abdi-Jalebi et al*.* proposed that the K^+^ doping in mixed perovskites can reduce non-radiative loss and light-induced ion migration by passivating the grain boundary and surface with potassium halide layers [130]. According to their point of view, the K^+^ doping would produce KI/KBr compounds that mainly distribute at grain boundary and surface (Fig. 7d), thus to immobilize the halide ions and vacancies, which is mutually contradictory to the above interstitial occupancy theory. Coincidentally, Kubicki et al*.* provided atomic-level characterization for the first time of K^+^-doped multi-cation and multi-anion perovskites [131]. The results unambiguously revealed that there is no evidence of K^+^ incorporation into the 3D perovskite, but the formation of non-perovskite KPbI_3_ (for iodide-based perovskites), mixed-K/Cs non-perovskite phase (for compositions containing Cs), or a mixture of KI and KBr (for mixed iodide-bromide perovskites). These secondary non-perovskite phases lead to variety in the composition of the pristine perovskite and in turn to shifts in XRD, PL, XPS and UPS spectra. In order to elucidate the mechanism of these K^+^ doping effects for PSCs, Zheng et al*.* combined ultrafast, time-resolved, and microspectroscopic techniques to dynamically monitor the changes of perovskite films under bias light illumination [132]. Different from the perovskite films without K^+^ doping, they detected a gradual halide ion substitution of Br^−^ by I^−^ under continuous illumination for the K^+^ doping case. And they proposed that illumination is essential to motivate the K^+^ passivation effect via forming immobile KBr-like compounds at the interfaces, leading to the reduction in interfacial defect density and suppression of ion migration (Fig. 7e), thus further results in improved PCE and negligible hysteresis. Besides, Wang et al*.* presented the formation of 2D K_2_PbI_4_ at the grain boundaries of WBG perovskite films, which can reduce the defect density and prevent ion migration, and thus, suppress non-radiative recombination and light-induced phase segregation [133]. Despite various theories, K^+^ does improve efficiency and photostability of PSCs. In addition to K^+^, Rb^+^ also shows beneficial effect in enhancing the crystallinity, enlarging grain size, reducing the excess PbI_2_ phase, as well as suppressing ion migration in WBG perovskite materials [134, 135].

**Fig. 8:**
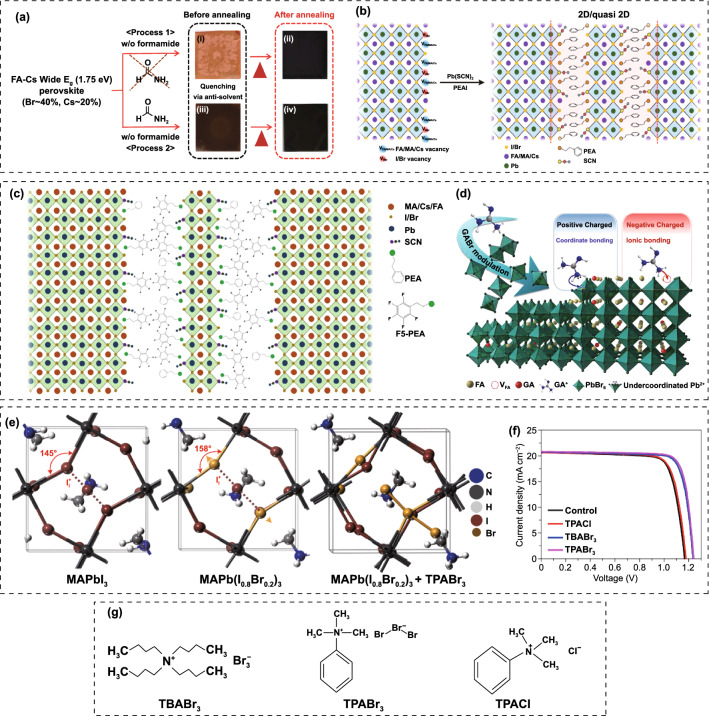
**a** FA-Cs WBG perovskite formation process without and with formamide additive. Reproduced with permission from Ref. [136]. Copyright 2018, Wiley–VCH. **b** Schematic illustration of defect passivation at perovskite grain boundaries (or surfaces) by PEA^+^ and SCN^−^. Reproduced with permission from Ref. [140]. Copyright 2019, Elsevier. **c** Illustration of perovskite structure based on mixed-cation 2D-PPA. Reproduced with permission from Ref. [144]. Copyright 2020, Wiley–VCH. **d** Schematic mechanism of GABr modulation on FAPbBr_3_ based PSCs. Reproduced with permission from Ref. [146]. Copyright 2022, Elsevier. **e** Geometrical structures of MAPbI_3_ and MAPb(I_0.8_Br_0.2_)_3_ with iodide interstitials, and the calculated geometrical structure of MAPb(I_0.8_Br_0.2_)_3_ with Br_3_^–^. **f** *J-V* curves of Cs_0.1_FA_0.2_MA_0.7_Pb(I_0.85_Br_0.15_)_3_ solar cells with additives of TPABr_3_, TBABr_3_ or TPACl, respectively. **g** Molecular structures of TBABr_3_, TPABr_3_ and TPACl. Reproduced with permission from Ref. [147]. Copyright 2022, Nature Publishing Group

Kim et al*.* added a formamide co-solvent into Cs-FA WBG perovskite precursor to control the crystallization aiming at suppressing light-induced phase segregation and hysteresis [136]. The highly polar formamide can bypass the yellow phase and induce direct formation of the black perovskite phase (Fig. 8a), thus reducing the trap density in film. Consequently, the optimized WBG PSCs (*E*_g_ ≈ 1.75 eV) afforded a high *V*_OC_ of 1.23 V and a PCE of 17.8% with reduced hysteresis. Meanwhile, the perovskite films demonstrated excellent photostability, thermal stability and long-term air stability. Tao et al*.* reported a stable ~ 1.73 eV bandgap MA-based WBG perovskite made from ionic liquid solvent, methylammonium acetate (MAAc) [137]. They revealed that the internal hydrogen bond (N–H···I and N–H···Br) environment in MAAc solvent over traditional DMF and DMSO solvents can stabilize the diffusion of halide ions, which favors the suppression of the light-induced halide segregation. Moreover, the hydrogen bonds can also enable excellent decoupling of the crystal nucleation and film growth, giving rise to a champion PCE of 20.59% with improved ambient light, thermal and air stability. Zhou et al*.* demonstrated that the photo-instability and *V*_OC_ loss can be addressed by combining crystallization control and grain boundary passivation [138]. They suggested that the Pb(SCN)_2_ additive can effectively enhance the crystallinity and grain size, while the excess FAX (X = I and Br) can passivate grain boundaries. The synergistic effects enable significantly improved carrier lifetime and suppressed photo-induced phase segregation. Xie et al*.* demonstrated that the Pb(SCN)_2_ additive can dramatically increase the grain size and inhibit the formation of pinholes in the MA-based WBG perovskite films [139]. And further introducing Cs^+^ cation into the precursor can effectively increase the solubility of PbI_2_, thus suppressing the aggregation of PbI_2_ particles induced by the Pb(SCN)_2_. The co-incorporation of Pb(SCN)_2_ and Cs^+^ cation contributed to high crystallinity and uniform MA_0.9_Cs_0.1_PbI_2_Br(SCN)_0.08_ perovskite films with a bandgap of 1.77 eV, yielding a PCE of 16.3%. Zhu’ group investigated the synergistic effect of PEAI and Pb(SCN)_2_ complementary additives in the WBG FA_0.65_MA_0.2_Cs_0.15_PbI_2.4_Br_0.6_ (1.68 eV) perovskite precursor (Fig. 8b) [140]. It is revealed that the incorporation of PEA^+^ spacer can result in the reduction in grain size and limit the charge transport. When there is only Pb(SCN)_2_ additive, the grain size was increased, but it also induced the formation of large amount of excess PbI_2_ phases. Interestingly, the co-incorporation of PEA^+^ and SCN^−^ can perfectly overcome the separate issues associated with each additive, giving rise to remarkably improved structural and optoelectronic properties of perovskite films: such as improved crystallinity and carrier mobility, reduced defect density and energetic disorder. The optimized solar cell delivered a PCE approaching 20%. In addition to SCN^−^ ions that can effectively increase grain size, the MACl additive is also widely used to improve the quality of perovskite film. Chen et al*.* presented that the MACl can significantly enlarge the grain size and improve the film morphology [141]. And further incorporation of MAH_2_PO_2_ can passivate the grain boundaries and suppress non-radiative recombination. The coupling of MACl and MAH_2_PO_2_ resulted in the WBG (1.64 ~ 1.70 eV) solar cells with improved photocurrent and reduced *V*_OC_ deficit, leading to a PCE of 25.4% for 2-T perovskite/silicon TSCs.

Long chain ammonium cations have been widely used to passivate grain boundary and surface of perovskite films by forming 2D structure or themselves [121–124, 142, 143]. Particularly, for WBG PSCs, this a promising strategy for simultaneously improving efficiency and photostability. Zhu and co-workers reported a 2D-passivator engineering for the WBG (~ 1.68 eV) perovskites for the first time [144]. The pentafluorophenethylammonium (F5PEA^+^) was introduced to partially replace the phenylethylammonium (PEA^+^) to form a robust non-covalent coordination between the two spacers (Fig. 8c). The WBG perovskite based on mixed ammoniums exhibited longer carrier lifetime, lower defect density and higher conductivity as compared with those using mono-cations, giving rise to enhanced PCE from 19.58% to 21.10% along with improved stability. Huo et al*.* compared the passivation effect of three organic bromide salts with different chain lengths including phenylammonium bromide (PhABr), phenmethylammonium bromide (PMABr) and phenethylammonium bromide (PEABr) on WBG (1.74 eV) PSCs [145]. PMABr with moderate chain length performs best in passivating defects and suppressing phase segregation. Xu et al*.* incorporated guanidinium bromide (GABr) into FAPbBr_3_ precursor to modulate the crystallization and heal the charged defects [146]. It is found that GABr can reduce the wettability of the precursor on the substrate, leading to suppressed heterogeneous nucleation and larger grain size, as well as released microstrain and strengthened lattice structure. Moreover, the ionized -NH_3_^+^ group and unsaturated N atoms could simultaneously heal negatively charged (A-site vacancy) and positively charged (uncoordinated Pb^2+^) defects, respectively (Fig. 8d). The FAPbBr_3_ solar cell gave a PCE of 8.92% with a *V*_OC_ of 1.639 V. Deep-level defects are recognized as the non-radiative recombination centers and severely sacrifice the *V*_OC_ of WBG PSCs. Yang et al*.* identified positive iodide interstitials as the deep-level defects in WBG perovskite films that limits the PCE improvement of PSCs [147]. In view of this, they dexterously introduced trimethylphenylammonium tribromide (TPABr_3_) as defect passivator into WBG perovskite precursor to inhibit the formation of iodide interstitials. The Br_3_^−^ is expected to occupy the halide sites or fill halide vacancies and leave two terminal Br atoms, which can inhibit the formation of iodide interstitials due to the space limit. As a result, the reduced charge recombination favored increased carrier collection distance and allowed 1-µm-thick WBG perovskite film on textured silicon cell, boosting the PCE of fully textured perovskite/silicon TSC to 28.6% (Fig. 8e, f). The tribromide additive can also suppress the light-induced phase segregation and thus enhance the photostability. Qin et al*.* reduced the non-radiative recombination through bulk defect passivation using chloro-formamidinium (ClFA^+^) additive [148]. The ClFA^+^ can simultaneously passivate iodine vacancy and Pb-I antisite defects, contributing to a 1.25 V *V*_OC_ and a FF of 83% for FA_0.6_MA_0.4_Pb(I_0.6_Br_0.4_)_3_ solar cells. And the 2-T perovskite/organic TSC delivered a high PCE up to 22.0%. Kim et al*.* proposed a anion engineering to carefully control the structural and electronic properties of 2D PbI_2_ passivation layers [149]. A mixture of thiocyanate (SCN) with iodine additive (PEAI_0.25_SCN_0.75_) was added into the FA_0.65_MA_0.2_Cs_0.15_PbI_2.4_Br_0.6_ precursor. As discussed before, SCN^−^ can promote the crystal growth and result in larger grain size, while the PEA^+^ apparently suppresses the crystal growth. Similarly, the coupling of I^−^ and SCN^−^ in PEAI_0.25_SCN_0.75_ additive significantly improves the crystallinity and enlarge the grain size, thus enhance the carrier lifetime and mobility. Most importantly, PEAI_0.25_SCN_0.75_ plays a critical role in modulating the desirable orientation of PbI_2_ frame work located at grain boundary and surface, favoring better passivation effect and superior stability. As a result, the single-junction PSC and 2-T perovskite/silicon TSC achieved PCEs of 20.7% and 26.7%, respectively.

**Fig. 9:**
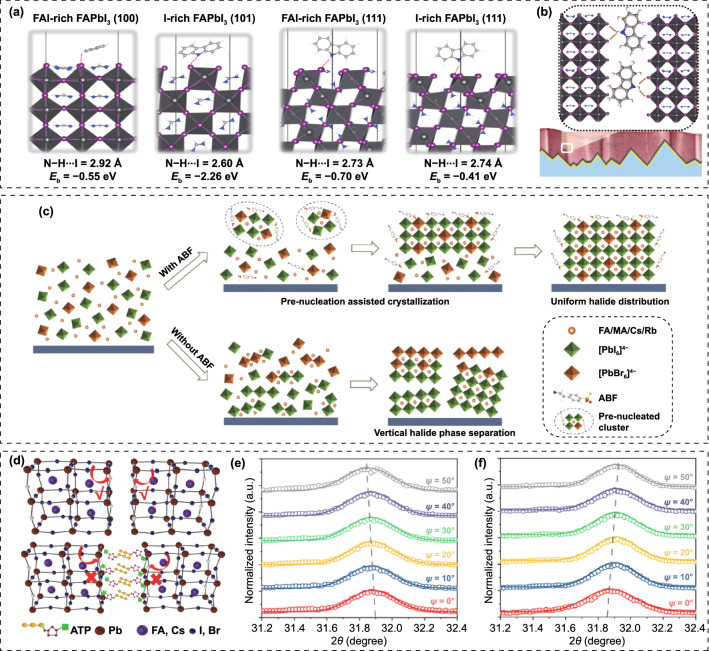
**a** Optimized interfacial structures for carbazole/FAPbI_3_ models with different surface orientations and terminations of FAPbI_3_, together with hydrogen bond lengths (red dashed lines) and binding energies (*E*_b_) between carbazole and perovskite. **b** Schematic illustration of interaction of carbazole with perovskite films. Reproduced with permission from Ref. [156]. Copyright 2021, Elsevier. **c** Schematic diagram of ABF pre-nucleation assisted crystallization process. Reproduced with permission from Ref. [157]. Copyright 2022, Wiley–VCH. **d** Schematic describing of interaction between ATP and perovskite. **e** XRD spectra in (001) planes of REF and ATP films before and after light soaking for 2 h. **f** W–H analysis for REF and ATP films before and after light soaking. Reproduced with permission from Ref. [69]. Copyright 2022, Wiley–VCH

Some organic small molecules and polymers bearing Lewis acid or base group are known for passivating defects at grain boundaries and suppressing non-radiative recombination [150–152]. Specific additives, such as imidazolium, theophylline and piperidinium, have also been reported to improve efficiency and stability via coordinate binding [153–155]. Liu et al. incorporated carbazole to passivate deep-level charged defects and suppress phase segregation of WBG perovskites [156]. Commonly, the ion migration starts from grain boundaries and is activated by strain, while the carbazole molecules can effectively stabilize the film surface through hydrogen bonding interactions with halides to impede the ion migration from the surface into the bulk, thus suppressing the phase segregation (Fig. 9a, b). Consequently, the carbazole-based WBG PSC gave a PCE of 20.2%, giving rise to 28.2% efficiency for 2-T perovskite/silicon TSCs. Zheng et al*.* demonstrated that homogenized crystallization of WBG perovskites is crucial for suppressing the initial vertical halide phase separation and reduce *V*_OC_ loss [157]. Hence, they introduced 4-(2-aminoethyl)-benzenesulfonyl fluoride hydrochloride (ABF) with multifunctional groups into the WBG perovskite precursor to induce downward homogenized crystallization. The ABF can accelerate the pre-nucleation but slowdown the crystal growth, which is conducive to the formation of large-size crystals. And it preferentially precipitated on the surface instead of Br compounds, holding back the aggregation of Br-rich phase and allowing a uniform halides distribution vertically across the film. The ABF-contained pre-nucleation on the surface serves as a template for the downward homogenized crystallization (Fig. 9c). Moreover, fluoride with strong electronegativity can effectively stabilize anions and cations, while the sulfonyl and ammonium groups are expected to passivate positively charged (halide vacancies) and negatively charged (A-site vacancies) defects, respectively. The reduced defect density and uniform halides distribution account for the improved photostability. The ABF-based 1.63 and 1.68 eV PSCs offered PCEs of 21.76% and 20.11%, respectively. Yao et al*.* reported that the detrimental I_2_ in the precursor would produce deep-level traps in final perovskite films [34]. A ammonium diethyldithiocarbamate (ADDC) additive is proven to reduce the I_2_ back to I^−^, thus reducing the deep-level defect density in the WBG perovskite film. Due to the decreased halide vacancies, the light-induced phase segregation can be effectively suppressed. The optimized WBG (1.77 eV) solar cell gave a PCE of 18.6% and a *V*_OC_ of 1.24 V along with great operation stability. Oliver et al*.* demonstrated that the incorporation of ionic additive (1-butyl-1-methylpiperidinium tetrafluoroborate) can effectively reduce non-radiative recombination in the bulk of WBG perovskite film and at interfaces contacting with the charge transporting layers [158]. The MA-free WBG (1.79 eV) PSCs yielded a PCE approaching 17%. Chen’ group focused on the strain investigation of the PSCs. The residual strain in the perovskite film is found to significantly affect the crystal and electronic structures, as well as carrier dynamics within the devices, which is mainly determined by the composition, substrates and temperature during the film preparation. They ascribed the photo-instability of WBG perovskites to the local tensile strain in the film, which can facilitate the halides segregation at grain boundaries in the I/Br mixed perovskites [69]. Through introducing adenosine triphosphate (ATP) additive, they achieved WBG perovskite film with compressive microstrain (Fig. 9d–f), which increases the energy barrier for ion migration and inhibits the photo-induced phase segregation for both A-site and X-site. The single-junction *n-i-p* solar cell demonstrated a champion PCE of 20.53%, yielding a PCE of 26.95% for monolithic perovskite/silicon tandems. Table 3 summarized the device performance of WBG PSCs employing additive engineering.

**Table 3:** Summary of WBG PSCs employing additive engineering

Perovskite	Additive	*E*_g_ (eV)	*V*_OC_ (V)	*J*_SC_ (mA cm^−2^)	FF (%)	PCE (%)	References
FA_0.85_MA_0.15_Pb(I_0.85_Br_0.15_)_3_	KI	1.60	1.167	22.99	76	20.32	[126]
Cs_0.05_(FA_0.85_MA_0.15_)_0.95_Pb(I_0.85_Br_0.15_)_3_	KI	1.59	1.132	22.95	79	20.56	[127]
FA_0.85_MA_0.1_Cs_0.05_PbI_2.7_Br_0.3_	KI	NA	1.140	22.16	74.3	18.77	[128]
FA_0.85_MA_0.15_PbI_2.7_Br_0.3_	KI	NA	1.136	21.52	72.7	17.11	[128]
Cs_0.06_FA_0.79_MA_0.15_Pb(I_0.4_Br_0.6_)_3_	KI	1.78	1.23	17.9	79	17.5	[130]
Cs_0.05_(FA_0.85_MA_0.15_)_0.95_Pb(I_0.85_Br_0.15_)_3_	KI	1.62	1.135	22.88	78	20.25	[132]
Cs_0.05_FA_0.79_MA_0.16_Pb(I_0.6_Br_0.4_)_3_	KI	1.75	1.26	19.19	76	18.38	[133]
FA_0.8_Cs_0.2_Pb(I_0.7_Br_0.3_)_3_	KI	1.71	1.185	19.6	79	18.3	[134]
FA_0.83_Cs_0.17_Pb(I_0.6_Br_0.4_)_3_	Formamide	1.75	1.23	18.34	79	17.8	[136]
MAPb(I_0.75_Br_0.25_)_3_	MAAc	1.73	1.22	20.85	81.11	20.59	[137]
MA_0.9_Cs_0.1_PbI_2_Br(SCN)_0.08_	Pb(SCN)_2_/CsI	1.77	1.15	17.4	81.4	16.3	[139]
FA_0.65_MA_0.20_Cs_0.15_Pb(I_0.8_Br_0.2_)_3_	PEAI/Pb(SCN)_2_	1.68	1.170	21.2	79.8	19.8	[140]
Cs_0.15_(FA_0.83_MA_0.17_)_0.85_Pb(I_0.8_Br_0.2_)_3_	MACl/MAH_2_PO_2_	1.64	1.15	NA	NA	19.3	[141]
FA_0.64_MA_0.20_Cs_0.15_Pb_0.99_(I_0.79_Br_0.2_)_3_	PEAI/F5PEAI	1.68	1.196	21.65	81.5	21.10	[144]
FA_0.75_MA_0.15_Cs_0.1_PbI_2_Br	PMABr	1.74	1.19	18.69	78.21	17.32	[145]
FAPbBr_3_	GABr	2.25	1.639	7.71	71	8.92	[146]
Cs_0.1_FA_0.2_MA_0.7_Pb(I_0.85_Br_0.15_)_3_	TPABr_3_	1.65	1.23	21.2	83.8	21.9	[147]
FA_0.6_MA_0.4_Pb(I_0.6_Br_0.4_)_3_	ClFA	1.75	1.25	16.9	83	17.6	[148]
FA_0.65_MA_0.20_Cs_0.15_Pb(I_0.8_Br_0.2_)_3_	Pb(SCN)_2_/PEAI/ PEASCN	1.68	1.2	NA	NA	20.7	[149]
FA_0.87_Cs_0.13_Pb(I_0.87_Br_0.13_)_3_	PEO	NA	1.098	19.87	75	16.46	[152]
Cs_0.05_FA_0.8_MA_0.15_Pb(I_0.75_Br_0.25_)_3_	Carbazole	1.68	1.22	20.6	81	20.2	[156]
Rb_0.05_Cs_0.05_(FA_0.75_MA_0.25_)Pb(I_0.75_Br_0.25_)_3_	ABF	1.68	1.207	20.98	79.45	20.11	[157]
Rb_0.05_Cs_0.05_(FA_0.83_MA_0.17_)Pb(I_0.83_Br_0.17_)_3_	ABF	1.63	1.18	22.56	81.76	21.76	[157]
NA	ADDC	1.77	1.25	20.11	81.21	20.41	[34]
FA_0.83_Cs_0.17_Pb(I_0.6_Br_0.4_)_3_	[BMP]^+^[BF4]^−^	1.79	1.22	NA	NA	17	[158]
Cs_0.22_FA_0.78_Pb(I_0.85_Br_0.15_)_3_	ATP	1.65	1.21	21.08	80.49	20.53	[69]

### Charge Transport Layers

The device structure of PSCs can be divided into *n-i-p* and *p-i-n* types according to the charge transport direction. To date, the certified PCE of *n-i-p* PSCs is slightly higher than that of *p-i-n* PSCs [159, 160]. The key reason could be the different CTLs employed in different devices. Compared with *p-i-n* PSCs, the superior energy level alignment between perovskites and CTLs in *n-i-p* PSCs contributes to higher efficiency. Though it is feasible to obtain high-efficiency single-junction WBG PSCs using *n-i-p* device structure, *p-i-n* WBG PSCs is preferred for perovskite-based TSCs. For *n-i-p* PSCs, Spiro-OMeTAD is the mostly used hole transport layers (HTLs) [3]. On the one hand, the hydrophilic properties of dopants like lithium bis(trifluoromethylsulfonyl)imide (LiTFSI) and tertbutylpyridine (tBP) in Spiro-OMeTAD lead to poor device stability in the air [161]. On the other hand, the thick Spiro-OMeTAD film (~ 200 nm) causes high parasitic absorption, thereby reducing the *J*_SC_ and sacrificing the efficiency of TSCs. Due to the lower parasitic absorption of thinner CTLs in *p-i-n* PSCs, TSCs with *p-i-n* structure have received more attention and are more promising to achieve higher PCE.

**Fig. 10:**
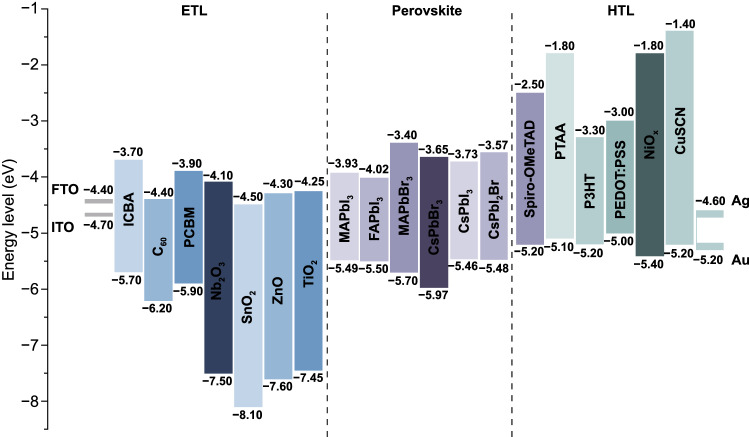
Energy level diagram of perovskites, ETLs, HTLs and electrodes in PSCs

CTLs play a critical role in charge transfer and extraction (Fig. 10) [74, 162–165]. For high-performance PSCs, the essential requirements of CTLs are high carrier mobility and well-matched energy level alignment with perovskites, which are directly related to the *J*_SC_, *V*_OC_ and FF of PSCs. Meanwhile, good chemical stability of CTLs is also important for long-term device stability. Furthermore, for perovskite-based TSCs, CTLs with good light transmittance is also crucial for better light management. The most commonly used electron transport layers (ETLs) include TiO_2_, SnO_2_ and fullerene derivatives, and the HTLs are NiO_x_, PTAA and Spiro-OMeTAD [166–172]. Whereas, the undesirable energy level alignment between WBG perovskites and these CTLs would induce serious interfacial non-radiative recombination, which is also the reason for substandard *V*_OC_ of WBG PSCs. Developing novel CTLs or modifying the existing CTLs to achieve better energy level matching is promising to reduce the *V*_OC_ loss. In the early studies of PSCs, TiO_2_ was the most widely used CTLs [86, 93]. However, the deposition of TiO_2_ layers requires high temperature (> 500 °C), which would damage the underlying rear cells in TSCs. Though low-temperature processed TiO_2_ nanoparticles and chemical bath deposition have been developed, the relatively low carrier mobility limits its further PCE improvement. Low-temperature processed SnO_2_ exhibits excellent charge transport property and gradually replace TiO_2_ in high-efficiency PSCs. Moreover, SnO_2_ possesses higher transmittance, making it very popular for transparent electrode in *p-i-n* TSCs [16, 17]. As discussed above, highly efficient TSCs prefer *p-i-n* structure. The main HTLs for WBG PSCs and TSCs are PTAA, NiO_x_ and self-assembled materials (SAMs) [147, 148, 173–175], while the ETLs are fullerene and its derivatives. Table 4 summarized the device performance of WBG PSCs with different CTLs.

**Table 4:** Summary of WBG PSCs with different CTLs

Perovskite	CTL	*E*_g_ (eV)	*V*_OC_ (V)	*J*_SC_ (mA cm^−2^)	FF (%)	PCE (%)	References
MAPbBr_0.8_I_2.2_	In-TiO_*x*_	1.75	1.21	15.8	77.9	14.9	[176]
MAPbBr_0.5_I_2.5_	In-TiO_*x*_	1.70	1.16	18.3	78.2	16.6	[176]
Cs_0.05_(MA_0.17_FA_0.83_)_0.95_Pb(I_0.83_Br_0.17_)_3_	2PACz	1.60	1.188	21.9	80.2	20.9	[183]
Cs_0.25_FA_0.75_Pb(I_0.85_Br_0.15_)_3_	2PACz	1.65	1.20	22.15	83.81	22.33	[175]
FA_0.6_MA_0.4_Pb(I_0.6_Br_0.4_)_3_	2PACz	1.75	1.25	16.9	83	17.6	[148]
Cs_0.12_FA_0.8_MA_0.08_PbI_1.8_Br_1.2_	2PACz	1.77	1.29	15	77.9	15.1	[186]
FA_0.8_Cs_0.2_Pb(I_0.7_Br_0.3_)_3_	MeO-2PACz/	1.75	1.21	19.3	86.5	20.2	[174]
FA_0.8_Cs_0.15_MA_0.05_Pb(I_0.82_Br_0.18_)_3_	Me-2PACz/ MeO-2PACz	1.65	1.221	21.5	83.3	21.9	[187]
Cs_0.05_(FA_0.77_MA_0.23_)_0.95_Pb(I_0.77_Br_0.23_)_3_	Me-4PACz	1.68	1.224	20.7	82.0	20.8	[107]
NA	NiO_*x*_/2PACz	1.65	1.14	23.19	80.6	21.31	[188]
Cs_0.05_(FA_0.84_MA_0.16_)_0.95_Pb(I_0.85_Br_0.15_)_3_	NiO_*x*_/SAM	1.59	1.10	21.9	77.4	18.6	[190]
FA_0.8_Cs_0.2_Pb(I_0.6_Br_0.4_)_3_	NiO_*x*_/Me-4PACz	1.79	1.33	18.06	84.2	20.2	[189]
FA_0.8_Cs_0.2_PbI_1.8_Br_1.2_	VNPB	1.77	1.23	17.0	79.8	16.7	[191]
Cs_0.1_FA_0.2_MA_0.7_Pb(I_0.85_Br_0.15_)_3_	PTAA	1.65	1.25	21.1	83	21.2	[147]
FA_0.65_MA_0.20_Cs_0.15_Pb(I_0.8_Br_0.2_)_3_	PTAA	1.67	1.195	21.11	81.87	20.64	[173]
Cs_0.1_MA_0.9_Pb(I_0.9_Br_0.1_)_3_	PTAA	1.65	1.167	21.0	82.0	20.1	[180]
MAPb(I_0.75_Br_0.25_)_3_	PTAA	1.68	1.20	NA	NA	18.05	[179]
(FA_0.83_MA_0.17_)_0.95_Cs_0.05_Pb(I_0.6_Br_0.4_)_3_	ICBA-tran3	1.71	1.20	19.7	77.5	18.3	[196]
MAPb(I_0.73_Br_0.27_)_3_	C_60_MC_12_	1.71	1.24	17.45	77	16.74	[197]
Cs_0.05_MA_0.15_FA_0.8_Pb(I_0.85_Br_0.15_)_3_	a-NbO_*x*_/C_60_	1.68	1.20	21.6	76.6	19.8	[198]
Cs_0.4_FA_0.6_PbI_1.95_Br_1.05_	C_60_/SnO_1.76_	1.78	1.23	16.5	78.9	16.0	[199]

**Fig. 11:**
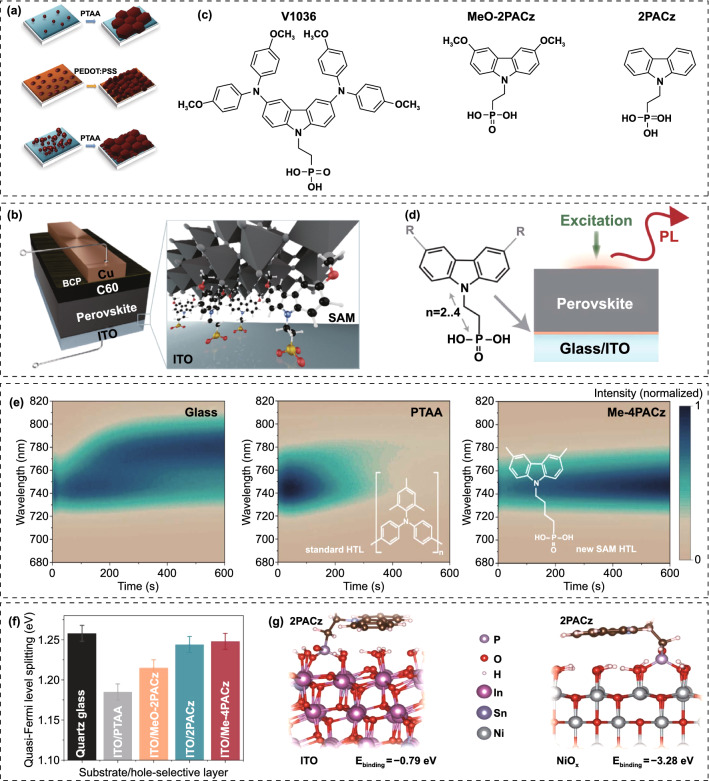
**a** Schematic illustration of nucleation and growth of grains on wetting and non-wetting hole transport layer surface. Reproduced with permission from Ref. [176]. Copyright 2016, Wiley–VCH. **b** Schematic of the device structure. The zoom-in visualizes how the SAM molecules attach to the ITO surface and therefore enable the hole selective contact to the perovskite above. **c** Chemical structure of the SAM molecules V1036, MeO-2PACz and 2PACz. Reproduced with permission from Ref. [183]. Copyright 2019, Royal Society of Chemistry. **d** Schematic of PL experiment and chemical structure of a general carbazole-based SAM. **e** Time-dependent PL spectra of perovskite films on different substrates. **f** Quasi-Fermi level splitting values of nonsegregated perovskite films on glass substrate and different hole-selective layers. Reproduced with permission from Ref. [107]. Copyright 2020, Science Publishing Group. **g** Theoretical modeling of 2PACz adsorption on ITO (left) and NiO_*x*_ (right) surfaces in the presence of surface hydroxyl groups based on DFT calculations. Reproduced with permission from Ref. [188]. Copyright 2022, Wiley–VCH

Hu et al*.* demonstrated that the non-wettable PTAA substrate is beneficial for crystal growth with large grain size [176]. Due to the hydrophobic surface, the nucleation would be suppressed and result in increased spacing between the adjacent nuclei, allowing outspread growth and larger grains (Fig. 11a). Large crystals mean less grain boundaries, which is beneficial for enhancing photostability of WBG perovskites. Some researchers would like to improve the conductivity of PTAA by doping with small molecules like F4-TCNQ. Zheng et al*.* reported the *p*-doping of PTAA to facilitate hole extraction for 2-T perovskite/silicon TSCs, making the device performance less sensitive to the thickness of PTAA [177]. The *p*-doping with ionic compound 4-isopropyl-4’-methyldiphenyliodonium tetrakis(penta-fluorophenyl-borate) (DPI-TPFB) dopant is activated by light-soaking treatment, resulting in nearly 22 times higher conductivity than that of pristine PTAA. The significantly enhanced conductivity translated into improved FF and boosted the PCE from 25.0% to 27.8%. Though Bush et al*.* demonstrated that PTAA performs better than NiO_x_ in reducing *V*_OC_ loss, it is difficult to deposit conformal and fully covered HTL on the textured silicon cells [178]. When the size of the pyramid is significantly decreased (< 1 µm), PTAA is also practicable for textured perovskite/silicon TSCs [179]. Chen et al*.* demonstrated a blade coating technique to produce the desired thin, uniform PTAA layer on the textured silicon surface through elevating the substrate temperature to 70 °C [180]. When the temperature changed to room temperature, PTAA layer is non-uniform even with the assistance of N_2_, which can be attributed to the accelerated evaporation of the toluene at 70 °C. As a result, the perovskite-silicon TSC achieved a PCE of 26%. Yang et al*.* revealed that more severe non-radiative recombination at PTAA/perovskite interface through the photoluminescence quantum yield (PLQY) measurement [147]. By introducing a LiF layer at PTAA/WBG perovskite interface, *V*_OC_ increased up to 1.25 V, which is the smallest *V*_OC_ deficit reported for WBG PSCs applied in perovskite/silicon TSCs. Compared with PTAA, NiO_x_ can be deposited on textured surface via electron beam or magnetron sputtering deposition techniques. Recently, self-assembled HTLs have made great progress on perovskite/silicon and perovskite/CIGS TSCs. These molecules can covalently bind to the conductive oxides like ITO and NiO_x_, and they can be prepared by spin-coating or dip-coating within wide processing windows. SAMs are made of small organic molecules, and thus, the chemical synthesis of most SAMs affords precise structures [181, 182]. Due to the ease of synthesis for carboxylic acid and phosphonic acid units, they were widely used in the SAMs. In addition, SAMs are of great potential to modify CTLs due to negligible parasitic optical losses, which can significantly improve the *J*_SC_ [183, 184]. For instance, MeO-2PACz ((2-(3,6-dimethoxy-9H-carbazol-9-yl)ethyl) phosphonic acid) can improve the *J*_SC_ of *p-i-n* PSCs by ~ 0.8 mA cm^−2^ compared with PTAA [183]. Similarly, a C_60_-SAM ETL improves the *J*_SC_ of *p-i-n* PSCs by absolute 1.2 mA cm^−2^ compared with TiO_2_ [185]. In 2019, Albrecht’ group reported a new generation of SAMs as the HTL for perovskite/CIGS TSCs (Fig. 11b) [183]. Compared with Spiro-OMeTAD and PTAA, SAMs are cheap, dopant-free, simple to process and intrinsically scalable. Moreover, they can offer the crucial advantage of conformal coverage on the rough CIGS film surface. Three different SAMs, (2-{3,6-bis[bis(4-methoxyphenyl)amino]-9H-carbazol-9-yl}ethyl)phosphonic acid (V1036), MeO-2PACz and [2-(9H-carbazol-9-yl)ethyl]phosphonic acid (2PACz) are employed (Fig. 11c), among which the 2PACz delivered the highest 20.9% efficiency for Cs_0.05_(MA_0.17_FA_0.83_)_0.95_Pb(I_0.83_Br_0.17_)_3_ solar cell and 23.26% efficiency for perovskite/CIGS TSCs. Later, they utilized methyl-substituted carbazole monolayer, named Me-4PACz ([4-(3,6-dimethyl-9H-carbazol-9-yl)butyl]phosphonicacid) as HTL in WBG PSCs (Fig. 11d), which enabled a high quasi-Fermi level splitting (QFLS) (Fig. 11f), allowing fast hole extraction and minimized non-radiative recombination. At the same time, the Me-4PACz substrate can also notably enhance the photostability under continuous illumination (Fig. 11e). The single-junction PSCs achieved a PCE of 20.8%, contributing to 29.15% efficiency for perovskite/silicon tandems with *V*_OC_ as high as 1.92 V [107]. Also, taking advantage of Me-4PACz, they further improve the certified PCE of perovskite/CIGS TSCs up to 24.2%, which is the record for this kind of TSCs [29]. In addition to reducing surface recombination on rigid indium tin oxide (ITO) substrates, SAMs are also suitable for flexible WBG. Lai et al*.* revealed that WBG perovskite deposited on a flexible polymer substrate shows better optoelectronic quality and uniformity by replacing PTAA with 2PACz, resulting a lower *V*_OC_ loss for a 1.77 eV flexible PSCs [186]. Particularly, in order to obtain uniform coverage of the 2PACz on the substrate, the solution should be left on the substrate for 60 s and repeated twice before spinning. As a result, flexible all-perovskite TSCs delivered a PCE of 23.8% with a remarkable *V*_OC_ of 2.1 V. Liu et al*.* deposited 1.65 eV bandgap perovskite on a HTL prepared with Me-2PACZ and MeO-2PACZ mixed solution to reduce recombination losses at perovskite/HTL interface, achieving the PCEs of 21.9% and 29.8% for WBG PSC and perovskite/silicon TSC, respectively [187]. Liu and co-workers developed a molecular-level nanotechnology by introducing an ultrathin hybrid NiO_x_/2PACz HTL atop the ITO recombination junction, serving as a vital pivot for the conformal deposition of perovskite layer [152] [188]. Compared with ITO/2PACz contact, the bonding reaction between NiO_x_ and 2PACz is energetically more favorable (Fig. 11g), which enables a uniform self-assembled 2PACz molecules on the fully textured surface, avoiding direct contact between perovskite and ITO to achieve minimal shunt loss. Recently, Chen et al*.* used NiO_x_/Me-4PACz as HTL to construct monolithic all-perovskite tandem, resulting a PCE > 27% with a record *V*_OC_ of 2.19 V [189].

Meanwhile, the hybrid HTL also resulted in obviously enhanced crystallinity and enlarged grain size. As a result, the fully textured perovskite/silicon TSCs delivered a record certified PCE of 28.84% on 1.2 cm^2^ masked area. Zheng et al*.* also employed NiO_x_/2PACz HTL for perovskite/silicon TSCs, and a PCE of 27.6% was obtained [190]. Tan’ group demonstrated increased *V*_OC_ and PCE of WBG PSCs by replacing commonly used PTAA with *in situ* cross-linked small molecule *N4*,*N4*′-di(naphthalen-1-yl)-*N4*,*N4*′-bis(4-vinylphenyl)biphenyl-4,4′-diamine (VNPB) [191]. The stronger interaction and lower defect density at the VNPB/perovskite interface contributed to the improved efficiency and stability of WBG PSCs, translating to PCEs of 24.9% and 25.4% for all-perovskite and perovskite-silicon TSCs, respectively. Moreover, by adopting NiO_x_/VNPB hybrid HTL for WBG PSCs, they achieved glaring progress for all-perovskite TSCs [56]. Besides, vacuum-deposited Spiro-TTB has also been utilized for TSCs [192–194]. In this case, the perovskite on Spiro-TTB should be prepared by two-step (vacuum-solution) or fully vacuum deposition method, because Spiro-TTB can be dissolved in DMF or DMSO solvent [195].

**Fig. 12:**
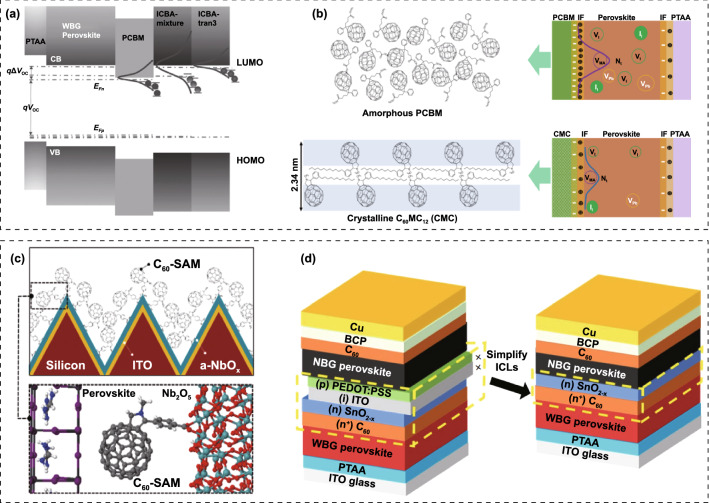
**a** Schematic illustration of how energy level and energy disorder of ETLs influences the device *V*_OC_. Reproduced with permission from Ref. [196]. Copyright 2017, Wiley–VCH. **b** Schematic illustration of the mechanism for performance enhancement in device with crystalline fullerene derivatives. Reproduced with permission from Ref. [197]. Copyright 2018, American Chemical Society. **c** Schematics of C_60_-SAM self-assembly process on Nb_2_O_5_. Reproduced with permission from Ref. [198]. Copyright 2021, Royal Society of Chemistry. **d** Schematic diagram of tandem devices based on typical structured and simplified ICLs. Reproduced with permission from Ref. [199]. Copyright 2020, Nature Publishing Group

To date, the role of fullerene (C_60_) in TSCs remains unchallenged. C_60_ and its derivatives (like PC_61_BM) are major ETLs for *p-i-n* PSCs. Considering that C_60_ can be the easily functionalized, it is feasible to synthesize derivatives with desirable energy levels to realize better contact with WBG perovskites. Lin et al*.* demonstrated that the indene-C_60_ bis-adduct (ICBA) with higher lowest-unoccupied molecular-orbital (LUMO) is beneficial for reducing *V*_OC_ loss for WBG PSCs [196]. However, the large energy disorder of ICBA will diminish the *V*_OC_ improvement due to the presence of many polymorphs. They presented that ICBA-tran3 isolated from ICBA-mixture exhibits the same high LUMO, but much smaller energy disorder and higher carrier mobility as compared with pristine ICBA-mixture (Fig. 12a). The application of ICBA-tran3 enabled enhanced efficiency up to18.5%. Khadka et al*.* synthesized long alkyl chain-substituted fullerene derivatives as ETL. The C_60_-fused *N*-methylpyrrolidine-*meta*-dodecyl phenyl (C_60_MC_12_) demonstrated high crystallinity compared to amorphous PC_61_BM, giving rise to more efficient electron transfer and lower perovskite/ETL interfacial defect (Fig. 12b) [197]. The 1.71 eV bandgap MAPbI_3-x_Br_x_ solar cell delivered a PCE of 16.74% with a *V*_OC_ of 1.24 V. Aydin et al*.* reported *n-i-p* 2-T perovskite/silicon TSCs by combining sputtered amorphous niobium oxide (a-NbO_x_) with ligand-bridged C_60_ as the underlying ETL and evaporated Spiro-TTB as HTL [198]. The C_60_ with a functionalized pyrrolidine tail was designed to achieve a conformal self-assembled monolayer to ensure efficient charge extraction and reduce parasitic absorption (Fig. 12c). The 2-T *n-i-p* perovskite/silicon TSCs gave a PCE of 27%, which is highest for TSCs with this polarity. Some studies revealed that C_60_ can be unintentionally *n*-doped by iodine ions from the perovskite films, resulting in elevated *E*_F_ and enhanced electron transportation property. Yu et al*.* found that the Sn/O ratio of SnO_2-x_ determines the electronic properties of the layer [199]. When 2-x = 1.76, the atomic layer deposition (ALD) processed SnO_2-x_ layer exhibits an ambipolar carrier transport property. Combining with *n*-doped C_60_, they simplified the interconnection layers (C_60_/SnO_2-x_/ITO/PEDOT:PSS) into C_60_/SnO_1.76_, both of which form good ohmic contacts with the corresponding sub-cells (Fig. 12d). With these merits, they achieved 24.4% efficiency for all-perovskite TSCs.

Though various fullerene derivatives have been developed, C_60_ is still preferred for highly efficient perovskite-based TSCs. Compared with C_60_, most of fullerene derivatives shows more parasitic absorption, which is fatal for current improvement in TSCs. Moreover, only C_60_ is able to be vacuum deposited, while other fullerene derivatives are solution-processed. Certainly, vacuum deposition technique allows dense and uniform ETL, which is also crucial for improving efficiency and stability of TSCs.

### Interface Engineering

PSCs present a typical sandwich structure, including perovskite layer, ETL/HTLs, charge buffer layers and electrodes. The multi-stacked structure means various interfacial contacts [200, 201]. The properties of these interfaces directly affect the charge transportation and non-radiative recombination, which are vital for the high-performance PSCs [202]. On the one hand, the non-matched energy levels and interfacial trap-assisted non-radiative recombination induce severe *V*_OC_ loss and thus limit the efficiency of WBG PSCs [60, 203, 204]. On the other hand, interfacial reactions induced by defects could also damage the device stability [205–207]. Therefore, interface engineering has been proven to be one of the most effective methods to achieve high-performance and long-term stable PSCs.

**Fig. 13:**
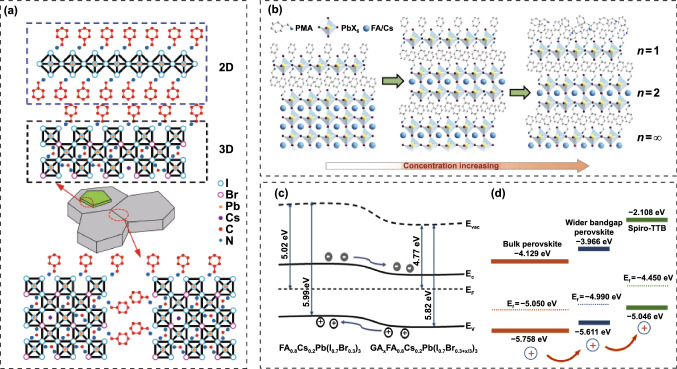
**a** Schematic of BA modification on the Cs_0.15_FA_0.85_Pb(I_0.73_Br_0.27_)_3_ thin film. Reproduced with permission from Ref. [211]. Copyright 2017, Wiley–VCH. **b** Schematic perovskite films treated with low, medium or high concentrations of PMABr/IPA solution. Reproduced with permission from Ref. [214]. Copyright 2020, Elsevier. **c** Energy level diagrams of perovskite film. Reproduced with permission from Ref. [217]. Copyright 2019, Elsevier. **d** Energy level diagram of perovskite and HTL after introducing the CsBr interface layer. Reproduced with permission from Ref. [194]. Copyright 2021, Wiley–VCH

Constructing interfacial 2D/3D heterostructures via long chain alkylamines is widely used to passivate defects, block ion migration, and suppress phase segregation of PSCs [208–210]. For instance, employing benzylamine (BA) post-treatment for 1.72 eV bandgap Cs_0.15_FA_0.85_Pb(I_0.73_Br_0.27_)_3_ perovskite, a 2D BA_2_PbI_4_ phase was obtained, which can effectively passivate defective regions and prevent decomposition or phase segregation [211]. Moreover, the formation of 2D BA_2_PbI_4_ (Fig. 13a) created an energy cascade with the underneath 3D perovskite, facilitating hole extraction while blocking electron transportation. As a result, the efficiency and ambient stability were greatly improved after the BA modification. Paetzold et al*.* introduced *n*-butylammonium bromide (BABr) into 1.72 eV bandgap Cs_0.17_FA_0.83_Pb(I_0.6_Br_0.4_)_3_ perovskite to construct a hybrid 2D/3D heterostructure at the interface toward the HTL, which mitigated non-radiative recombination in the perovskite film [212]. The work function increased from -4.27 to -3.94 eV with BABr treatment, suggesting better energy level alignment and thus faster carrier extraction at perovskite/HTL interface. Eventually, the BABr-modified PSCs demonstrated a stable power output conversion efficiency of 19.4% and a *V*_OC_ up to 1.31 V, which was the highest *V*_OC_ reported for WBG PSCs. To further confirm whether it is better to introduce 2D perovskite by surface post-treatment or bulk incorporation, Catchpole et al*.* introduced BABr with different strategies to obtain a mixed-dimensional system [213]. It was found that *Ruddlesden–Popper* quasi-2D perovskite phase formed on the surface can effectively passivate the defects and optimize the electronic structure at the surface of the 3D perovskite, resulting in longer carrier lifetime and higher efficiency. In contrast, incorporating BABr into the precursor solution formed pure 2D perovskite phase, negatively affecting the crystallinity and electronic structure, leading to poor device performance. Though different large cation salts have been successfully used for post-treatment, there is still a lack of understanding of the impact of these large cations on the interfacial morphology. Huang et al*.* precisely controlled the surface structure and thickness of the 2D perovskites via phenylmethylamine bromide (PMABr) post-treatment for a 1.74 eV bandgap perovskite film (Fig. 13b) [214]. It is found that the microstructures at the interface were strongly depended on the concentration of the post-treatment solution. Meanwhile, the Br ions in PMABr can passivate the halide vacancies. Recently, thiophene-based quasi-2D *Ruddlesden–Popper* (*R-P*) and *Dion-Jacobson* (*D-J*) perovskites have demonstrated preferable photovoltaic performances than their counterparts based on PEA cations due to the improved carrier mobility from the strong polarity of sulfur atoms. Fang et al*.* reported 2-thiopheneethylammonium chloride (TEACl) post-treatment for FA_0.8_Cs_0.2_Pb(I_0.8_Br_0.2_)_3_ perovskite (1.68 eV) film, where a 2D interlayer of TEA_2_PbI_4_ with *n* = 1 was formed between the absorber and ETL [215]. With this desired hierarchical 2D/3D structure, the efficiency and stability were simultaneously improved through passivating the electron and hole traps, increasing the activation energy of the ion migration and lowering the dark saturation current density of the device. The WBG *n-i-p* PSCs with TEACl treatment showed a PCE of 20.31%. It has been demonstrated that surface post-treatment using ammonium halides can effectively reduce *V*_OC_ deficit in WBG PSCs but at present the mechanism is still unclear. He et al*.* reported a phenethylammonium bromide (PEABr) post-treatment strategy with different annealing temperatures for precisely tailoring the phase purity of 2D perovskites on 3D WBG perovskite, passivating surface defects and optimizing surface electric field [216]. After PEABr post-treatment, a pure *n* = 1 2D perovskite phase formed at 60 °C on the top of a 1.77 eV WBG perovskite, which significantly suppressed non-radiative recombination thereby decreased the *V*_OC_ deficit. As a result, the 1.77 eV WBG PSC gave a *V*_OC_ of 1.284 V, which is the lowest *V*_OC_ deficit (0.486 V) among WBG PSCs with a bandgap higher than 1.75 eV.

In addition to construct 2D phases, tuning the electronic properties of perovskite film surface is also a effective approach to improve device performance. Yan et al*.* applied guanidinium bromide (GABr) to create graded perovskite homojunction on FA_0.8_Cs_0.2_Pb(I_0.7_Br_0.3_)_3_ perovskite (1.75 eV), where a composition of GA_x_FA_0.8_Cs_0.2_Pb(I_0.7_Br_0.3+x/3_)_3_ was formed between the absorber and HTL [217]. GABr-treated perovskite films demonstrated more *n*-type property with decreased work function (Fig. 13c). The *V*_OC_ of GABr-treated *p-i-n* PSCs increased from 1.12 to 1.24 V with superior photostability. Later, in order to investigate the mechanism of defects passivation with large ammonium cations, they further combined the microscopic probing of localized electrical properties, thermal admittance spectroscopy (TAS) measurements and first-principles calculations of defect migration [218]. TAS measurement showed that PEAI-based device exhibited a higher *E*_a_ of 0.905 eV as compared with 0.680 eV for the control, indicating the significantly suppressed ion migration, which is in good agreement with the results of the theoretical calculation. Finally, the 1.73 eV bandgap PSCs achieved an efficiency of 19.07% with a *V*_OC_ deficit of 0.48 V. Catchpole et al*.* studied the effects of three bromide-containing alkylammonium organic cations with different chain lengths on defect passivation [219]. The results suggested that long chain alkyl organic cations were more suitable for defect passivation by changing the electronic structure of perovskite films. The optimized PSCs based on FA_0.75_MA_0.15_Cs_0.1_Rb_0.05_PbI_2_Br exhibited a PCE of 19.1% with excellent moisture and photostability. Li et al*.* inserted CsBr between HTL and perovskite by thermal evaporation, creating a graded perovskite absorber by reacting with residual PbI_2_ [194]. TRPL results showed that CsBr interface layer can accelerate the hole transport from perovskite to HTL through better aligned energy levels (Fig. 13d). With device optimization, perovskite/c-Si tandem cell reached an efficiency of 27.48% with a long-term stability over 10,000 h in N_2_ glovebox.

**Fig. 14:**
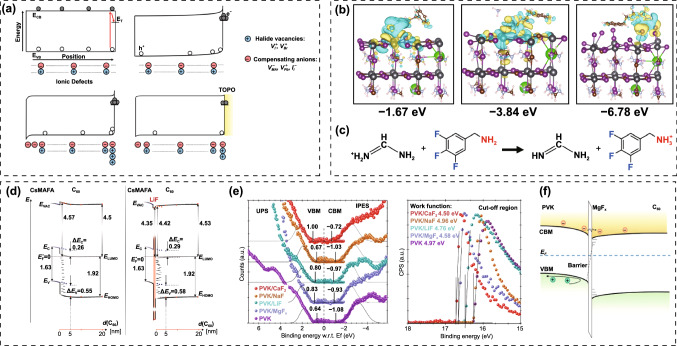
**a** Annotated band diagrams produced from drift–diffusion simulations. Reproduced with permission from Ref. [223]. Copyright 2018, American Chemical Society. **b** Electron density differences for the PhenH^+^ molecule on the PbI_2_, I-deficient and Pb-deficient surfaces and corresponding binding energies. Reproduced with permission from Ref. [224]. Copyright 2021, Elsevier. **c** Proton-transfer reaction between FA cation and TFBA from Ref. [205]. Copyright 2022, Wiley–VCH. **d** Energy level alignment at the CsMAFA/C_60_ interface with and without LiF interlayer. Reproduced with permission from Ref. [227]. Copyright 2022, Wiley–VCH. **e** Valence band and photoelectron cut-off region of the perovskite and perovskite/interlayers **f** Energy level diagram of the perovskite/C_60_ interface with MgF_x_ insertion layer. Reproduced with permission from Ref. [13]. Copyright 2022, Science Publishing Group

For the Br-rich WBG perovskites, the light-induced phase segregation primarily originates from the ionic migration via halide vacancies, which mainly occurs at grain boundaries and film surface [220–222]. McGehe et al*.* studied the relationship between the surface modification and light-induced halide segregation by introducing trioctylphosphine oxide (TOPO) onto CH_3_NH_3_PbI_2_Br [223]. They proposed possible drift–diffusion mechanisms for halide segregation by detecting the rate of halide segregation, providing a direct link between surface modification and photo-induced trap formation (Fig. 14a). Wolf et al. introduced phenformin hydrochloride (PhenHCl) containing both electron-rich and electron-poor moieties to simultaneously passivate defect and suppress the light-induced phase segregation [224]. As shown in Fig. 14b, DFT calculations for Cs_0.13_MA_0.13_FA_0.74_Pb(I_0.81_Br_0.19_)_3_ corroborate that PhenHCl can effectively passivate different perovskite surfaces, including perfectly cleaved lead iodide (PbI_2_) surface, iodide-deficient surface and lead-deficient surface. Absolute PL imaging showed that PhenHCl passivation effectively suppresses phase segregation, in agreement with bandgap imaging of samples. As a result, PhenHCl-based 1.68 eV bandgap PSCs afforded a PCE of 20.5% with no *V*_OC_ loss after more than 3000 h of thermal stability test at 85 °C. The perovskite/silicon tandem solar cell achieved an efficiency of 26.5%. In addition to post-treatment, surface gradient passivation via anti-solvent can also achieve effective defect passivation and simplify the preparation process. Liu et al*.* proposed a in situ defect passivation strategy by introducing 3,4,5-trifluorobenzylamine (TFBA) into ethyl acetate (EA) as anti-solvent for the film preparation, where proton-transfer from formamidinium (FA) and methylammonium (MA) to TFBA (Fig. 14c) was observed [205]. The gradient distribution of TFBA significantly suppressed non-radiative recombination both at the surface and grain boundaries, as well as inhibiting phase segregation. Most of works focused on improving the *V*_OC_ of WBG PSCs, but ignore that PCE is also dramatically limited by the fill factor. Non-ideal charge transport between the perovskite absorber and electrodes can increase the series resistance, which limit the device performance. Therefore, Ho-Baillie et al*.* introduced a cation-diffusion-based double-sided interface passivation scheme by GuBr to improve the fill factor for 1.75 eV bandgap PSCs [174]. They showed that partial cation diffusion from the Gu-based passivation layers into the perovskite can suppress the shallow traps in the bulk, while the rest of the Gu cations remained at interfaces to passivate the deeper surface trap-states. As a result, they demonstrated a record FF of 86.5% and a PCE of 20.2%. This provided new insights for future passivation strategies based on ionic diffusion to prepare highly efficient and stable WBG PSCs.

The contact layer interfaces also play an essential role in determining the device performance of PSCs, especially for TSCs. Both the energy level mismatch and the trap states at interfaces strongly affect the charge transportation [225]. To date, C_60_ and PC_61_BM are commonly applied as ETLs for *p-i-n* PSCs. Moreover, easy vapor deposition processing technique and low parasitic absorption loss make evaporated C_60_ the most popular ETL for perovskite-based TSCs. However, energy level mismatch and severe charge recombination at the perovskite/C_60_ interface limit the performance of TSCs. In this case, interlayers between perovskite and C_60_ are commonly adopted. Neher et al*.* reported an ultrathin LiF layer between perovskite and C_60_ to reduce the interfacial recombination loss by 35 meV [226]. Besides, inserting an ultrathin LiF layer at the perovskite/C_60_ interface for 1.68 eV bandgap *p-i-n* PSCs leads to a maximum voltage of 1.234 V and a PCE of 20.8% [107]. However, the mechanism of LiF passivation is still poorly understood. Korte et al*.* proposed a field effect passivation by using ultra-sensitive near-UV photoelectron spectroscopy (CFSYS) to study the electronic interface formation and energy level alignment at the perovskite/C_60_ interface [227]. The incorporation of a LiF layer at the interface can reduce the hole concentration by a mild dipole effect and probably the presence of fixed charges (Fig. 14d). As a result, the defect density in the first monolayers of C_60_ was effectively reduced, leading to improved *V*_OC_. Similarly, Snaith et al*.* introduced a LiF interface treatment for a MA-free 1.79 eV bandgap perovskite to reduce non-radiative recombination. The *n-i-p* PSCs with LiF treatment showed a *V*_OC_ of up to 1.22 V and a PCE approaching 17% [158]. However, on the one hand, the deliquescent behavior and high ion diffusivity of Li salts may reduce device stability. Recently, Wolf et al. investigated a series of metal fluorides to instead of LiF as the interlayers at the perovskite/C_60_ interface [13]. Ultraviolet photoemission spectroscopy (UPS) and low-energy inverse photoemission spectroscopy (LE-IPES) showed that work function systematically shifts toward smaller values, and the valence band maximum of the perovskite was lowered relative to its Fermi level, implying that the metal fluorides caused a downward band bending at the perovskite interface, which is benefit for more efficient electron extraction (Fig. 14e, f). As a result, the monolithic perovskite/silicon TSCs with a MgF_x_ contact displacer reached a *V*_OC_ of 1.92 V, giving rise to a certified PCE of 29.3% with ~ 95% remain after 1000 h damp-heat testing. Table 5 summarized the device performance of WBG PSCs employing interface engineering.

**Table 5:** Summary of WBG PSCs employing interface engineering

Perovskite	Modifying material	*E*_g_ (eV)	*V*_OC_ (V)	*J*_SC_ (mA cm^−2^)	FF (%)	PCE (%)	References
FA_0.83_Cs_0.17_Pb(I_0.76_Br_0.24_)_3_	BABr	1.65	1.18	NA	NA	19.2	[208]
Cs_0.05_FA_0.79_MA_0.16_Pb(I_0.67_Br_0.33_)_3_	HABr	1.72	1.31	19.60	77.10	19.8	[165, 209]
Cs_0.2_FA_0.8_Pb(I_0.82_Br_0.15_Cl_0.03_)_3_	ODADI	1.66	1.23	20.79	82.28	21.05	[210]
Cs_0.15_FA_0.85_Pb(I_0.75_Br_0.27_)_3_	BA	1.72	1.24	19.83	73.7	18.13	[211]
Cs_0.17_FA_0.83_Pb(I_0.6_Br_0.4_)_3_	BABr	1.72	1.31	19.3	78	19.8	[212]
Rb_0.05_Cs_0.095_MA_0.1425_FA_0.7125_PbI_2_Br	*n*-BABr	1.72	1.269	18.9	76.2	18.3	[213]
FA_0.8_Cs_0.2_Pb(I_0.7_Br_0.3_)_3_	PMABr	1.74	1.204	19.84	78	18.51	[214]
FA_0.8_Cs_0.2_Pb(I_0.8_Br_0.2_)_3_	TEACl	1.68	1.19	20.94	81.8	20.31	[215]
FA_0.8_Cs_0.2_Pb(I_0.7_Br_0.3_)_3_	GABr	1.75	1.24	17.92	81.90	18.19	[217]
FA_0.8_Cs_0.2_Pb(I_0.7_Br_0.3_)_3_	PEAI	1.73	1.25	19.48	78.9	19.07	[218]
FA_0.75_MA_0.15_Cs_0.1_Rb_0.05_PbI_2_Br	*n*-OABr	1.72	1.282	18.9	78.8	19.1	[219]
FA_0.9_Cs_0.1_PbI_2.87_Br_0.13_	CsBr	1.64	1.082	19.59	80.34	17.03	[194]
Cs_0.15_FA_0.85_Pb(I_0.71_Br_0.29_)_3_	Al_2_O_3_	1.72	1.22	15.4	73.4	13.8	[220]
Cs_0.09_FA_0.77_MA_0.14_Pb(I_0.84_Br_0.16_)_3_	PEAI	1.64	1.17	18.60	81.1	17.7	[221]
Cs_0.15_MA_0.15_FA_0.7_Pb(I_0.8_Br_0.2_)_3_	PhenHCl	1.68	1.22	NA	NA	20.5	[224]
Cs_0.22_FA_0.78_PbI_2.55-x_Br_0.45_Cl_x_	TFBA	1.68	1.204	20.72	81.73	20.39	[205]
FA_0.8_Cs_0.2_Pb(I_0.7_Br_0.3_)_3_	GuBr	1.75	1.21	19.3	86.5	20.2	[174]
(CsPbI_3_)_0.05_[(FAPbI_3_) _0.89_(MAPbBr_3_)_0.11_]_0.95_	LiF	NA	1.17	21.7	78.6	20.0	[226]
Cs_0.05_(FA_0.77_MA_0.23_)_0.95_Pb(I_0.77_Br_0.23_)_3_	LiF	1.68	1.224	20.7	82.0	20.8	[107]
FA_0.83_Cs_0.17_Pb(I_0.6_Br_0.4_)_3_	LiF	1.79	1.22	NA	NA	17	[158]
Cs_0.05_FA_0.8_MA_0.15_Pb(I_0.755_Br_0.255_)_3_	MgF_*x*_	1.69	1.226	20.58	81.1	20.46	[13]

### Preparation Method

Preparation techniques for perovskite films include one-step and two-step solution methods, vapor-assisted solution and full evaporation methods [228, 229]. Considering the diversity and complexity of WBG perovskites, different preparation techniques might facing different issues and produce different perovskite films. For example, compared with one-step solution method, it is more difficult to tailor the bandgap of perovskites via two-step solution and evaporation methods, as well as realize complete conversion from raw materials to perovskite films. Therefore, optimizing preparation methods of WBG PSCs are also quite important. To date, most highly efficient PSCs are still at lab-cell level, and the active areas are smaller than 1 cm^2^, while the large-area cells demonstrate decreased PCE mainly due to the increased trap states. Upscaling fabrication is of prime importance for PSCs commercialization. Slot-die coating, blade coating, inkjet printing, roll-to-roll printing and evaporation are familiar fabrication methods for making PSC modules [230–232]. Here, we will emphatically introduce the preparation methods of WBG perovskites in 2-T tandem conditions.

**Fig. 15:**
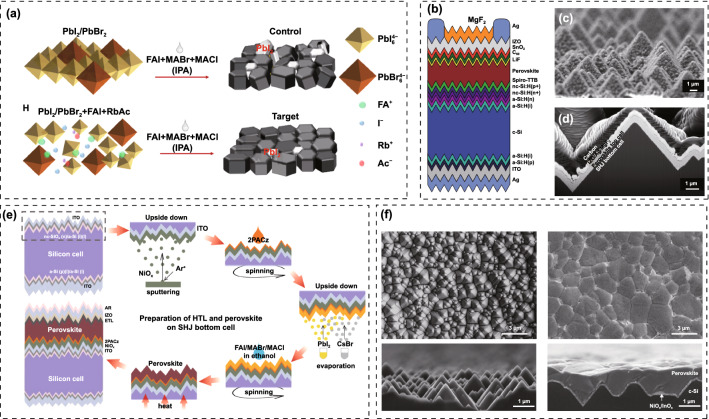
**a** Schematic diagram of two-step preparation method. Reproduced with permission from Ref. [238]. Copyright 2022, American Chemical Society. **b** Schematic view of a fully textured monolithic perovskite/SHJ tandem. **c** Secondary electron SEM image of the perovskite layer. **d** Cross section of the full perovskite top cell deposited on the SHJ bottom cell. Reproduced with permission from Ref. [193]. Copyright 2018, Nature Publishing Group. **e** Schematic illustration of the processing procedures of NiO_x_/2PACz hybrid HTL and perovskite on fully textured bottom SHJ cell. Reproduced with permission from Ref. [188]. Copyright 2022, Wiley–VCH. **f** SEM top-view and cross-sectional images of the textured *c*-Si and *c-*Si/perovskite layers. Reproduced with permission from Ref. [243]. Copyright 2020, Science Publishing Group

Most of perovskite-based TSCs present active area ~ 1 cm^2^, which are realized by one-step spin-coating method with anti-solvent process. Whereas, the Br-rich perovskites undergo faster crystallization than that of normal-bandgap perovskites, which might induce uncontrollable crystal growth, and lead to more trap states and inferior reproducibility. Therefore, modulating the crystallization of one-step processed WBG perovskites is critical for high-performance perovskite-based TSCs [233–235]. For two-step solution method, PbI_2_/PbBr_2_ layer is firstly deposited, followed by reacting with FAX/MAX (X = I, Br, Cl) isopropanol solution. This method is capable of depositing thick perovskite films, while adjusting the bandgap to desirable values is more complicated than one-step method. Though it has been successfully applied to normal-bandgap (< 1.6 eV) PSCs [2, 236], it has not been widely employed for WBG PSCs and 2-T TSCs [101]. Liu et al*.* successfully fabricated WBG perovskites with a bandgap of 1.63–1.65 eV using two-step solution method via optimizing the solution composition and the rotate speed of the second step. Solar cells gave a PCE of 20.35% with a high fill factor of 81.53% [237]. Chen et al*.* introduced formamidinium iodide (FAI) and rubidium acetate (RbAc) into the PbI_2_/PbBr_2_ complex to create temperate particle size for nucleation site, thus facilitating the diffusion of FAI/MABr/MACl [238]. While the control PbI_2_/PbBr_2_ film demonstrated better crystallinity and larger grain size, which would definitely impede the diffusion of organic salts, resulting in incomplete conversion. Consequently, the FAI + RbAc additives contributed to preferentially (100)-oriented growth of the WBG perovskite film (Fig. 15a), giving rise to a PCE of 27.64% for 2-T perovskite/silicon TSCs.

Vacuum-deposition of perovskite films is a solvent-free process, which is beneficial for better film stability. Longo et al*.* reported fully vacuum-deposited MAPb(Br_x_I_1−x_)_3_ perovskite films with bandgaps of 1.72 and 1.87 eV via regulating the deposition rates of the different halide raw materials [239]. And the solar cells using these perovskite films afforded PCEs of 15.9% and 10.5%, respectively. Bolink’ group developed a 2-T MAPbI_3_/MAPbI_3_ TSC via vacuum-deposition method and achieved a extremely high *V*_OC_ of 2.30 V, yielding a PCE of 18.02% [240]. In order to reduce the light reflection, texturing process is widely employed for highly efficient silicon solar cells. Whereas, the pyramid textured surface makes it quite difficult to deposit conformal and full-covered perovskite layer upon it. Therefore, combining evaporation and solution process is put forward to prepare perovskite film on textured silicon solar cells, that is, a conformal PbX_2_ layer is deposited by evaporation, and then spin-coating organic salts solution. Sahli et al. reported a fully textured perovskite layer by co-evaporating a PbI_2_ and CsBr compounds before spin-coating an organohalide solution (FAI, FABr) [193]. The co-evaporation of CsBr can produce a porous PbI_2_ layer to facilitate the diffusion of FAI and FABr, achieving the conformal growth of perovskite film directly on the micrometer-sized pyramids of textured silicon cell (Fig. 15b–d). In addition, all the charge transport layers and buffer layers are deposited by evaporation technique, realizing a fully textured 2-T perovskite/silicon TSCs. The perovskite and silicon sub-cells presented fantastic current-matching, that are 20.1 and 20.3 mA cm^−2^, respectively, contributing to a certified PCE of 25.2%. Albrecht’ group introduced MAI to stabilize the FAPbI_3_ during the co-evaporation process [241]. Impressively, by optimizing the MAI/FAI ratio, solar cells maintained 100% of the initial efficiency after constant operation with 1000 h. And the fully textured 2-T perovskite/silicon TSC offered a PCE of 24.6%. Furthermore, they also investigated the influence of the MAI purity on device performance. Usually, the low-purity MAI contains hydroiodic acid (HI) and hypophosphorous acid (H_3_PO_2_) [242]. They found that the evaporated of low-purity MAI would go straight to the substrate due to the low sublimation temperature, while the evaporation of high-purity MAI requires high sublimation temperature, resulting in a cloud-like MAI vapor in the chamber. The impurities (HI and H_3_PO_2_) in the MAI would lead to poor stability of perovskite films. Liu and co-workers adopted NiO_x_/2PACz HTL, co-evaporation of PbI_2_ and CsBr followed with spin-coating FAI/MABr/MACl ethanol solution for the preparation of perovskite top-cell, achieving a highest certified efficiency of 28.84% for the fully textured perovskite/silicon TSCs (Fig. 15e) [188].

**Fig. 16:**
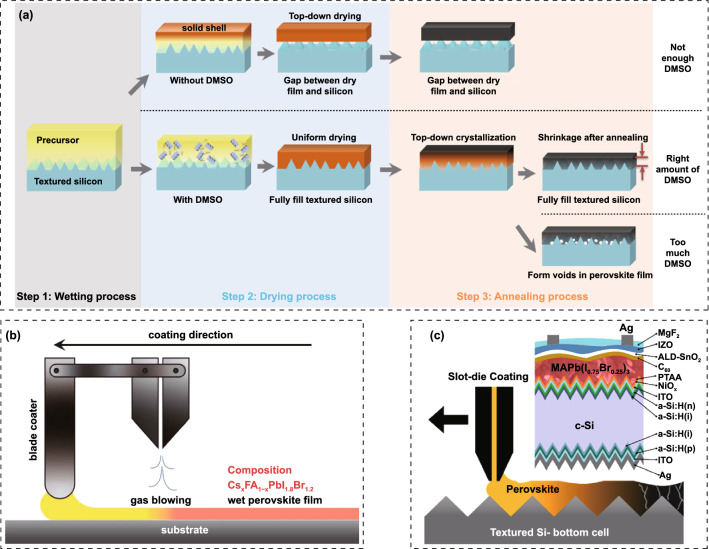
**a** Schematic depiction of the evolution of a perovskite layer on a textured surface. Reproduced with permission from Ref. [180]. Copyright 2020, Elsevier. **b** Schematic illustration of gas-assisted blade coating. Reproduced with permission from Ref. [110]. Copyright 2022, Science Publishing Group. **c** Schematic illustration of slot-die-coating. Reproduced with permission from Ref. [179]. Copyright 2020, American Chemical Society

In addition to the vacuum deposition technique, solution-processed perovskite on textured silicon cells with small pyramids (< 1 μm) is also realizable. In this case, high concentration of perovskite precursor using multi-step spin coating process is commonly required to achieve thick perovskite film and complete coverage on the pyramids. Hou et al. prepared a 1.65–1.75 M Cs_0.05_MA_0.15_FA_0.8_PbI_2.25_Br_0.75_ precursor solution and adopted three consecutive spin-coating steps of 300, 1,500, and 5,000 rpm, which allowed a micrometer-thick perovskite film on the fully textured silicon cell (Fig. 15f), yielding a 25.7% efficiency for TSCs [243]. Huang’s group demonstrated a nitrogen-assisted blading process to deposit both conformal hole transport layer and perovskite layer that fully covers the silicon cell textured with pyramids less than 1 μm in height [180]. The thickness of the blade-coated perovskite film can be tuned through the concentration of the perovskite precursor, the gap distance between the substrate and blade, and the speed of blade-coating. A crucial challenge is to deposit dense perovskite films with desirable thicknesses that are intimately contact with the textured silicon surface. In view of this, they tuned the ratio of DMSO/Pb in precursor considering that DMSO can coordinate with the perovskite to form intermediate phases and thus be expected to control the crystallization. For the DMSO-free system, the dying process starts at the air/solution interface as the solvent evaporates from the top surface, quickly forming a solid shell. The shell can serve as template to grow downward as the remaining precursor dries. When the last 2-methoxyethanol (2-ME) solvent evaporates and no precursor remains to fill its volume, the voids would be formed between the dry film and textured wafer. Fortunately, the DMSO can prohibit the quick formation of solid top shell due to its high boiling point and strong ability of coordinating with perovskite to form intermediate phase, which would allow 2-ME to evaporate and suppress the formation of voids. However, too much DMSO in the precursor could also cause voids formation, which can be explained by the shrinkage of the annealed film as due to the release of DMSO from the intermediate phase (Fig. 16a). Only for moderate DMSO concentration (25% in their experiment) can compensate the volume reduction by perovskite diffusion upon DMSO departure, thus achieving a dense WBG perovskite film on textured surface after annealing. As a result, they achieved a PCE of 26.2% for the perovskite/silicon TSC. Tan and co-workers found that the crystal nucleation rate of CsFA-based (Cs_x_FA_1-x_PbI_1.8_Br_1.2_) WBG perovskite films can be well controlled by finely tuning the Cs ratio, combining with a gas-assisted blade-coating technique (Fig. 16b) [110]. Interestingly, the crystallinity and crystal orientation of the blade-coated films were strongly related to the Cs ratio, which showed weak impact on spin-coated films. The champion Cs_0.35_FA_0.65_PbI_1.8_Br_1.2_ solar cell offered a PCE of 17.2% with an *V*_OC_ of 1.266 V, contributing to a 21.7% efficiency for 20 cm^2^ area all-perovskite tandem module. Subbiah et al*.* reported slot-die-coated MAPbI_2.25_Br_0.75_ PSCs for three different conditions: using the acetonitrile (ACN) + MA gas (dissolved in methanol) (MA(MeOH)) solvent precursor; using the ACN + MA(MeOH) solvent with L-α-phosphatidylcholine (LP) additive; and using the ACN + MA(MeOH) solvent with the LP additive and cysteine hydrochloride (Cys.HCl) surface treatment [179]. Consequently, the slot-die-coated 2-T perovskite/silicon TSC (Fig. 16c) achieved a PCE of 23.8% using the third recipe. Table 6 summarized the device performance of WBG PSCs with representative preparation method. It can see that most of high-performance WBG PSCs are made from one-step spin-coating method, which is not suitable for upscaling fabrication of TSCs. Though blade-coating and slot-die have been widely used for normal-bandgap PSCs, their application in perovskite-based TSCs needs further exploration.

**Table 6:** Summary of WBG PSCs with representative preparation method

Perovskite	preparation method	*E*_g_ (eV)	*V*_OC_ (V)	*J*_SC_ (mA cm^−2^)	FF (%)	PCE (%)	References
FAMACsPb(I_0.8_Br_0.2_)_3_	One-step solvent	1.67	1.195	23.06	79.32	21.85	[235]
Cs_0.22_FA_0.78_PbI_2.55_Br_0.45_	One-step solvent	1.67	1.217	20.18	83.16	20.42	[99]
Cs_0.22_FA_0.78_Pb(I_0.85_Br_0.15_)_3_	One-step solvent	1.67	1.19	20.33	81.7	19.76	[100]
Cs_0.1_FA_0.2_MA_0.7_Pb(I_0.85_Br_0.15_)_3_	One-step solvent	1.65	1.25	21.1	83	21.2	[147]
Cs_0.05_FA_0.8_MA_0.15_Pb(I_0.75_Br_0.25_)_3_	One-step solvent	1.68	1.218	20.2	80.6	20.2	[156]
Cs_0.05_MA_0.15_FA_0.8_Pb(I_0.85_Br_0.15_)_3_	One-step solvent	1.68	1.200	21.6	76.6	19.8	[198]
Cs_0.15_MA_0.15_FA_0.7_Pb(I_0.8_Br_0.2_)_3_	One-step solvent	1.68	1.22	NA	NA	20.5	[224]
Cs_0.05_(FA_0.77_MA_0.23_)_0.95_Pb(I_0.77_Br_0.23_)_3_	One-step solvent	1.68	1.224	20.7	82.0	20.8	[107]
Cs_0.05_FA_0.8_MA_0.15_Pb(I_0.755_Br_0.255_)_3_	One-step solvent	1.69	1.226	20.58	81.1	20.46	[13]
NA	Two-step solvent	1.63	1.13	22.11	81.53	20.35	[237]
FA_x_Cs_1-x_Pb(I_y_Br_1-y_)_3_	Two-step solvent	1.63	1.20	21.82	80.40	21.02	[238]
FA_0.5_MA_0.38_Cs_0.12_PbI_2.04_Br_0.96_	Two-step solvent	1.69	1.162	16.17	69.64	13.09	[101]
MAPbI_2.4_Br_0.6_	Two-step solvent	1.72	1.02	17.5	73.7	13.1	[87]
NA	Vacuum + Solution	1.65	1.14	23.19	80.6	21.31	[188]
FA_0.9_Cs_0.1_PbI_2.87_Br_0.13_	Vacuum + Solution	1.64	1.082	19.59	80.34	17.03	[194]
Cs_x_FA_1−x_Pb(I,Br)_3_	Vacuum + Solution	1.6	1.046	18.4	59.6	11.51	[193]
MAPb(Br_0.2_I_0.8_)_3_	Vacuum deposition	1.72	1.119	17.3	82.3	15.9	[239]
MAPb(Br_0.5_I_0.5_)_3_	Vacuum deposition	1.87	1.207	11.4	76.9	10.6	[239]
Cs_0.1_MA_0.9_Pb(I_0.9_Br_0.1_)_3_	Blade coating	1.65	1.167	21.0	82.0	20.1	[180]
Cs_0.35_FA_0.65_PbI_1.8_Br_1.2_	Blade coating	1.80	1.266	16.8	80.9	17.2	[110]
MAPb(I_0.75_Br_0.25_)_3_	Slot-die coating	1.68	1.20	NA	NA	18.05	[179]

## Outlook

### Crystallization Control

High-quality perovskite film is the guarantee of high-performance PSCs. Though there are plenty of crystallization modulation strategies surrounding normal-bandgap and all-inorganic perovskites, effective approaches put forward for organic–inorganic hybrid WBG perovskites are relatively few. Both heavy Cs and Br ions doping can arise ultrafast crystallization, leading to inferior crystallinity, amounts of defects and irreversible microstrain, thereby significantly affecting efficiency and stability, especially for upscaling fabrication. Here, we suggest efforts can be carried out from the following aspects:

1. Figuring out the colloidal chemistry of WBG perovskite precursor, such as species of colloidal complexes, solute coordination interaction, and colloidal particle sizes [244].
2. Forming intermediate phase via suitable additives to slow down the crystallization rate of WBG perovskites, and achieve uniform and large crystals [245, 246].
3. Optimizing the wettability of substrates, surface of wet precursor film and annealing temperature to modulate the surface free energy for nucleation and crystal growth.
4. Pointedly bonding with halogens to improve the distribution uniformity and immobility of anions.

### Composition Optimization

Although the bandgap of perovskites is the same, their compositions may be diverse, which might induce quite different crystal and electronic structures, thus further affecting the intrinsic optoelectronic properties. Different combinations can also influence the colloidal chemistry and crystallization process to influence the film quality, like grain size, defects and lattice stress. In addition, composition is also vitally important for the stability issues, including structural, light, air and thermal stability. Suggestions on composition engineering of WBG perovskites can be taken into account:

1. Exploring the effect of composition on carrier mobility and lifetime, formation energy of defects, as well as ion migration barrier.
2. Harmonizing the ratio of A and X-site ions to eliminate or minimize the size mismatch that could induce additional lattice stress.
3. Developing low-temperature processed and high-performance inverted all-inorganic PSCs to promote the application of photostable CsPbI_3-x_Br_x_ perovskites in tandem devices.

### Defect Passivation

Defects are deadly for efficiency and stability improvement. Trap-assisted non-radiative recombination is responsible for *V*_OC_ loss, while ion migration along halide vacancy results in phase segregation. Though great progress has been made focusing on defect passivation, the corresponding mechanism kept fuzzy, and most of works are based on trial-and-error method. In order to reduce *V*_OC_ loss and improve stability, we believe that:

1. Further illuminating the defect type like vacancies and anti-site defects, positively or negatively charged. This is vital for providing more pointed and effective defect passivation.‘’
2. Grain boundaries, buried interface and film surface assemble amounts of different defects, simultaneously modifying these positions to achieve better passivation effect deserves further exploration.
3. Multi-site passivation provided by multi-functional molecules are also necessary.

### Charge Transport Optimization

It is critical to develop novel CTLs to minimize the energy level mismatch between WBG perovskite and CTLs, thus to effectively enhance the charge transportation and reduce *V*_OC_ loss. Meanwhile, for underlying CTLs, it should be able to conformally cover the textured pyramids of silicon cell or rough surface of CIGS cell, and they are also expected to bond with perovskite to passivate buried interface. Furthermore, since PSCs demonstrate a typical sandwich structure, which contains various interfaces, like perovskite/CTLs interfaces, CTLs/electrodes interfaces, etc., optimizing these interfaces is also conducive to improving interfacial energy alignment, facilitating charge transfer and reducing interfacial non-radiative recombination.

### Upscaling Fabrication

To date, high-efficiency WBG PSCs were successfully achieved by spin-coating method, and it is urgent to transfer the small-area solar cells into upscaling modules [247, 248]. Whereas, the ultrafast crystallization of WBG perovskites makes it challenge for depositing high-quality perovskite films with large-area and low defect density. Therefore, crystallization control and defect passivation become extremely important for upscaling fabrication. In addition, since WBG perovskites facing tandem application, large-area fabrication of CTLs and transparent electrodes cannot be ignored. Magnetron sputtering, electron beam, atomic layer deposition techniques and so on are expected to be widely used.

### Stability

The extremely high efficiency of perovskite-based TSCs makes it promising for commercialization upon the stability issues can be well addressed. Besides the above mentioned strategies via controlling composition and crystallization to prepare high-quality perovskite films to improve film stability, the chemical stability of CTLs or buffer layers are also crucial for device stability [249–251]. They are expected to eliminate the diffusion of ions from metal electrodes or halide ions from perovskite films. Moreover, ALD-deposited SnO_2_ and NiO_x_ have also been proven to greatly strength the device stability. Certainly, industrialized encapsulation technique is quite necessary to protect the device from water and oxygen erosion. Once the long-term stability is addressed, the perovskite-based TSCs will be ready for commercialization.

## Conclusion

In this review, we summarized recent progress of WBG PSCs. The compositions, additives, charge transport layers, interfaces and preparation methods are carefully discussed. The effect of crystallization and crystal structure on device performance are highlighted. Challenges including *V*_OC_ deficit, stability and module fabrication are still the key concerns for WBG PSCs. And we gave an critical outlook on the strategies toward the highly efficient, long-term stable and large-area WBG PSCs for tandem application. As the increasing interest for perovskite-based TSCs around the world, we believe these challenges will be overcome, and the performance of WBG PSCs will continue to surprise us.
